# Salivary Cystatin D Interactome in Patients with Systemic Mastocytosis: An Exploratory Study

**DOI:** 10.3390/ijms241914613

**Published:** 2023-09-27

**Authors:** Simone Serrao, Cristina Contini, Giulia Guadalupi, Alessandra Olianas, Greca Lai, Irene Messana, Massimo Castagnola, Giulia Costanzo, Davide Firinu, Stefano Del Giacco, Barbara Manconi, Tiziana Cabras

**Affiliations:** 1 Department of Life and Environmental Sciences, University of Cagliari, 09124 Cagliari, Italy; simone.serrao@unica.it (S.S.); giulia.guadalupi@unica.it (G.G.); olianas@unica.it (A.O.); grecalai710@gmail.com (G.L.); bmanconi@unica.it (B.M.); 2 Istituto di Scienze e Tecnologie Chimiche “Giulio Natta”, Consiglio Nazionale delle Ricerche, 00168 Rome, Italy; imessana53@gmail.com; 3 Proteomics Laboratory, European Center for Brain Research, (IRCCS) Santa Lucia Foundation, 00168 Rome, Italy; maxcastagnola@outlook.it; 4 Department of Medical Sciences and Public Health, 09124 Cagliari, Italy; giuliacostanzo14@gmail.com (G.C.); davide.firinu@unica.it (D.F.); delgiacco@unica.it (S.D.G.)

**Keywords:** mastocytosis, cystatin D, protein interactions, human saliva, mass spectrometry

## Abstract

Mastocytosis, a rare blood disorder characterized by the proliferation of clonal abnormal mast cells, has a variegated clinical spectrum and diagnosis is often difficult and delayed. Recently we proposed the cathepsin inhibitor cystatin D-R_26_ as a salivary candidate biomarker of systemic mastocytosis (SM). Its C_26_ variant is able to form multiprotein complexes (mPCs) and since protein–protein interactions (PPIs) are crucial for studying disease pathogenesis, potential markers, and therapeutic targets, we aimed to define the protein composition of the salivary cystatin D-C_26_ interactome associated with SM. An exploratory affinity purification-mass spectrometry method was applied on pooled salivary samples from SM patients, SM patient subgroups with and without cutaneous symptoms (SM+C and SM−C), and healthy controls (Ctrls). Interactors specifically detected in Ctrls were found to be implicated in networks associated with cell and tissue homeostasis, innate system, endopeptidase regulation, and antimicrobial protection. Interactors distinctive of SM−C patients participate to PPI networks related to glucose metabolism, protein S-nitrosylation, antibacterial humoral response, and neutrophil degranulation, while interactors specific to SM+C were mainly associated with epithelial and keratinocyte differentiation, cytoskeleton rearrangement, and immune response pathways. Proteins sensitive to redox changes, as well as proteins with immunomodulatory properties and activating mast cells, were identified in patients; many of them were involved directly in cytoskeleton rearrangement, a process crucial for mast cell activation. Although preliminary, these results demonstrate that PPI alterations of the cystatin D-C_26_ interactome are associated with SM and provide a basis for future investigations based on quantitative proteomic analysis and immune validation.

## 1. Introduction

Mastocytosis is a rare hematopoietic neoplasm caused by an anomalous accumulation of clonal, morphologically, and phenotypically abnormal mast cells, which can accumulate in one or more organs [1]. Usually, mast cells accumulate in the skin, gastrointestinal tract, bone marrow, spleen, liver, and lymph nodes. The World Health Organization (WHO) classify the different forms of mastocytosis according to mast cell localization [2,3]. Cutaneous mastocytosis (CM) is characterized by mast cell accumulation in the skin; it is typical of pediatric age and has a good prognosis, the skin lesions often disappear spontaneously during puberty. In systemic mastocytosis (SM), mast cells invade and accumulate in at least one extra-cutaneous organ, such as the bone marrow or gastrointestinal tract [1,2,3]. SM includes indolent SM (ISM), hepatology neoplasm (SM-AHN), aggressive SM (ASM), and mast cell leukemia (MCL). In advanced SM, mast cell infiltration leads to organ damage and failure [1,2,3]. SM forms are typical of adulthood and rare in children [4]. A key role in the development of mastocytosis seems to be played by c-KIT, a tyrosine kinase receptor expressed on the mast cell surface [5]. Among the several mutations of the *c-KIT* gene found in SM patients [6], D816V is associated with the uncontrolled proliferation and activation of mast cells [3,7]; this leads to an abnormal release of inflammatory mediators, including histamine, interleukin 3 and 16, tumor necrosis factor-α, and proteolytic enzymes, as tryptase, which cause local and distal inflammation and allergic reactions [8,9]. Except for the tryptase level, no other molecular markers are available for mastocytosis diagnosis, which is instead based on the evaluation of clinical manifestations, skin biopsies, and eventually genetic analyses after bone marrow biopsy [2,3]. Novel non-invasive diagnostic biomarkers are strongly needed, especially to classify the different forms of mastocytosis. Saliva is a body fluid suitable for this purpose because of its non-invasive and easy collection, which does not require healthcare personnel [10,11]. Furthermore, the dynamic range of protein concentration in saliva reduces the challenge of detecting low abundant proteins with respect to plasma. Saliva contains proteins/peptides of both glandular and non-glandular origin, released by leucocytes present in the gingival crevicular fluid and by epithelial cells of mucosa, deriving from the blood and central nervous system [12]. Recently, our proteomic study on saliva from patients affected by SM and CM revealed significant quantitative changes, compared to a healthy control group, of peptides and proteins involved in mouth homeostasis, oral and systemic innate immunity, and inflammatory response [13]. Among them, we found particularly interesting the trend of cystatin D des1-5 and des1-8, two proteoforms deriving from N-terminal cleavage of the protein. They were more abundant in SM patients without cutaneous symptoms (SM−C) than in patients with both systemic and cutaneous manifestations (SM+C); interestingly, the level of the des1-5 proteoform correlated with tryptase concentration [13]. Cystatin D is an inhibitor of cathepsins B, H, L, and S [14], involved in innate immunity and inflammation [15,16]. The two alleles of cystatin D gene encode protein variants with either arginine or cysteine residue at position 26, named here cystatin D-R_26_ and D-C_26_, respectively [17]. The expression of these two variants in the Western population was established to be 55% for C_26_ variant and 45% for R_26_ variant [18]. In our first study [13], Cystatin D-R_26_ was detected in 54% and 58% of SM patients and healthy subjects, respectively, and almost all the SM−C patients exhibited the variant R_26_ (88%), while the SM+C patients showed a percentage (44%) similar to that expected based on the study of Balbín [18].

Several proteoforms of cystatin D-R_26_, but none of the C_26_ variant, have been identified in human saliva through top-down proteomic platforms able to examine intact proteoforms [13,19,20]. A previous preliminary study performed on whole salivary samples from healthy adults using immunoprecipitation coupled to MS analysis demonstrated that the cystatin D-C_26_ was associated with several other proteins in a high molecular weight multi-protein complex (mPC) [21]. The study of mPCs and protein–protein interaction (PPI) networks is considered a key factor with increasing interest in the characterization of new protein markers, the understanding of the molecular mechanisms of the diseases, the identification of novel therapeutic targets, and thus, the development of new therapeutic approaches [22]. Indeed, most human diseases are multifactorial and may be the consequences of either multiple genetic or epigenetic changes, infections, inflammation, or metabolic imbalance. In this context, the involved proteins must be considered part of complex PPI networks, able to connect and interact with several other proteins and also forming mPCs, which may be functionally and structurally affected by the disease. In this way, the impact of a disease-related mutation is not restricted to a specific protein, but extends to the entire set of interactors to which that protein is related [22]. Considering all these premises, as well as the significance of the regulation of cathepsins’ activity on allergic and inflammatory processes and the diagnostic potential of cystatin D, already suggested for mastocytosis in our previous study [13], this exploratory study aimed to investigate the cystatin D-C_26_ interactome in the saliva of patients with different forms of SM and to evaluate qualitative differences and similarities between SM patients and healthy controls (Ctrls). To this purpose, we applied an affinity purification-mass spectrometry (AP-MS) method, which relies on co-immunoprecipitation (CoIP) experiments coupled to bottom-up proteomic analysis, to highlight differences in the composition of cystatin D-C_26_ mPC, underlying different PPI networks and biological implications, between SM patients and healthy subjects.

## 2. Results

**Figure 1:**
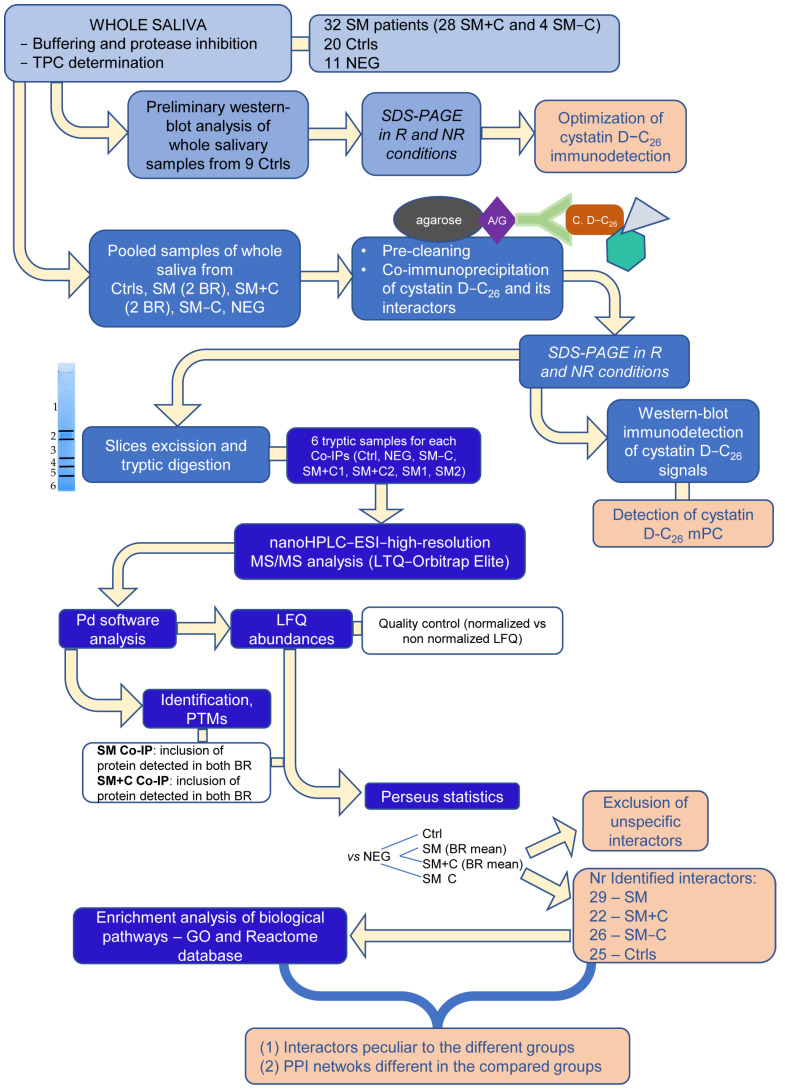
Workflow describing the experimental processes flowed in this AP-MS study for the qualitative characterization of the cystatin D-C_26_ multiprotein complex (mPC) in the groups under study: healthy controls (Ctrls), systemic mastocytosis patients (SM), and subgroups of SM patients with and without cutaneous symptoms (SM+C and SM−C). The main results are indicated in orange squares. TPC, total protein concentration; NEG, negative control group; R and NR, reducing and non-reducing conditions; BR, biological replicates; Pd, Proteome Discoverer software (version 2.5); LFQ, label-free quantification; PTMs, post-translational modifications; Co-IP, co-immunoprecipitated samples; and PPI, protein–protein interaction.

The AP-MS approach used in this study to detect and characterize possible salivary cystatin D-C_26_ multiprotein complex (mPC) in SM and HC individuals was based on CoIP with a specific Ab recognizing the variant C_26_. This procedure is illustrated in the workflow shown in Figure 1.

### 2.1. Explorative Western Blot Analysis of Cystatin D-C_26_ in Salivary Samples

A preliminary, exploratory Western blot analysis of whole salivary samples from nine healthy control subjects was performed under non-reducing (NR) and reducing (R) conditions, to optimize immune detection of the C_26_ variant of cystatin D (Supplementary Figure S1a,b). In agreement with previous results [21], the positive signal of cystatin D-C_26_ Ab was evident in non-reducing samples at molecular weight (MW) > 250 kDa (Supplementary Figure S1a) and not detected under R conditions (Figure S1b). The signal, attributed to a mPC associated with cystatin D-C_26_, showed as resistant to SDS denaturing conditions but not to reducing conditions. In addition, a positive signal at 10 kDa > MW < 15 kDa was revealed under both NR and R conditions. It was attributed to cystatin D-C_26_, agreeing with its expected average mass value of 13,858.60 Da.

### 2.2. Co-Immunoprecipitation of Cystatin D-C_26_-Bound mPC

**Figure 2:**
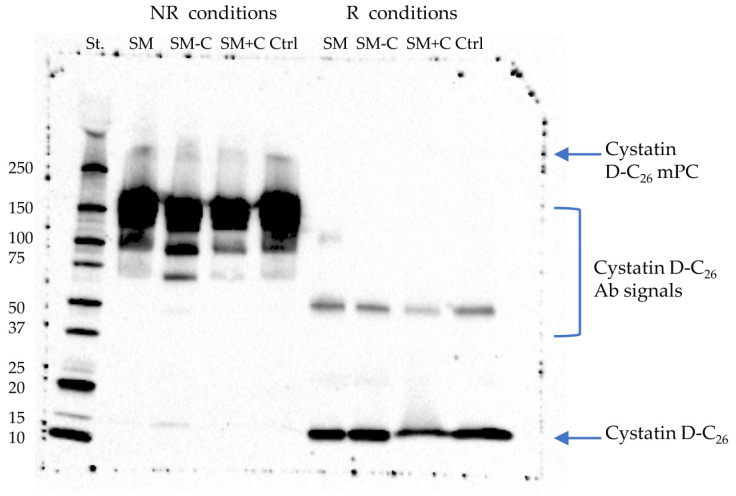
Immunodetection of cystatin D-C_26_ by Western blot analysis. Molecular weight standards (St) and Co-IPs from SM, SM−C, SM+C, and Ctrl groups treated under NR conditions, and the corresponding Co-IPs treated under R conditions.

Co-immunoprecipitated (Co-IPs) complexes were obtained from pools of whole saliva samples collected from each group under study, SM, SM−C, SM+C, and Ctrls. Western blot analysis of Co-IPs, highlighted a signal positive to cystatin D-C_26_ Ab at MW > 250 kDa under NR condition (Figure 2), the signal disappeared in R conditions where the strong positive signal at 10 kDa > MW < 15 kDa was attributed to cystatin D-C_26_ released from the mPC following the reducing treatment. The same signal, while much less intense, was detected in NR samples; this suggests that it is likely that a small amount of cystatin D-C_26_ was bound with a non-SDS resistant linkage in the high MW mPC.

Other intense signals at 150 kDa in NR samples and at 50 kDa in R samples were attributed to the entire Ab and to the heavy chain of the Ab, respectively.

### 2.3. High-Resolution MS/MS Analysis of Co-IP Samples

To identify the proteins co-immunoprecipitated with cystatin D-C_26_ and thus define the composition of the mPCs, and to highlight any differences among the groups of patients (the whole SM group and the subgroups SM+C and SM−C) and the Ctrls, we applied an AP-MS procedure based on the bottom-up high-resolution (HR)-MS/MS analysis of the co-immunoprecipitated proteins separated by SDS-PAGE under R conditions. As shown in Supplementary Figure S2a,b, each excised gel slice was treated separately.

Co-IPs from the SM1 and SM2 pools, as well as from the SM+C1 and SM+C2 pools, were separately analyzed by (HR)-MS/MS as biological replicates.

**Figure 3:**
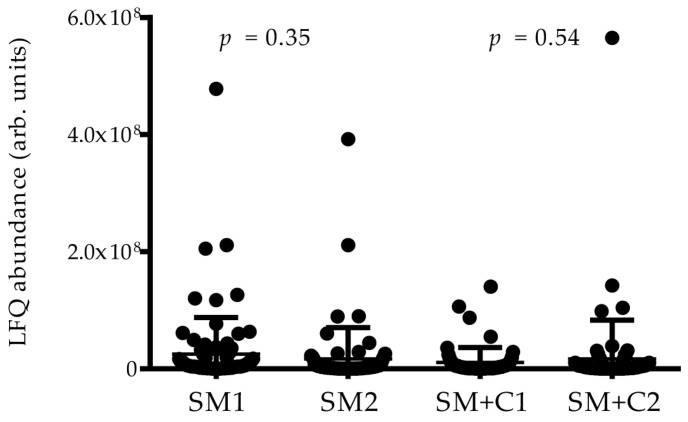
Plot with LFQ abundance distribution for proteins/peptides identified in the two SM replicates and in the two SM+C replicates. *p* values provided by statistical comparison with an unpaired *t*-test are reported.

The (HR)-MS/MS analysis of the Co-IPs led to the identification of the following: 77 proteins in Co-IP from the Ctrl pool; 85 and 69 proteins in Co-IPs from SM1 and SM2 pools, respectively; 66 proteins from SM−C pool; and 60 and 78 proteins from SM+C1 and SM+C2 pools, respectively, excluding contaminant proteins (keratins, Ig, histones, and hemoglobin) [23,24]. Finally, 66 proteins were identified in NEG Co-IP, which was used as a negative control group to exclude unspecific interactors. To obtain more robust statistical results against the NEG Co-IP, a quality control was performed comparing the protein LFQ abundances of Co-IPs from patient and Ctrls (Supplementary Figure S3a,b). The LFQ abundances, normalized according to the total peptide amount and their variances, appeared more comparable among the different Co-IPs (Figure S3b) with respect to the non-normalized data (Figure S3a); thus, normalized data were used. This result ensured that any possible difference measurable among Co-IPs were not attributable to uneven extraction of peptides from SDS-PAGE gels or to other experimental steps. Moreover, similar LFQ abundances of proteins identified in the Co-IPs from SM replicates, as well as from SM+C replicates were found (Figure 3). To further reduce the biological variability, only proteins detected in both SM1 and SM2 replicates (63 identifications) have been considered for comparison with NEG Co-IP; their LFQ abundances were calculated and used by Perseus software (version 1.6.15.0). Similarly, this was performed for the 56 proteins identified in both SM+C1 and SM+C2 replicates. Consequently, in the rest of the Result and Discussion sections we referred only to the SM and SM+C groups. The proteins found to be enriched in patient groups and in Ctrls with respect to NEG Co-IP were considered to be interactors of cystatin D-C_26_, and thus components of the mPC revealed in our samples. The results of the comparison against NEG Co-IP are reported in Supplementary Table S1, where the excluded proteins are in italics.

The number of proteins identified as components of the mPC associated with cystatin D-C_26_, comprising cystatin D-C_26_ itself, was reduced to 29 in Co-IP from SM group, 22 in Co-IPs from SM+C, 26 in Co-IP from SM−C groups, and 25 in Co-IP from the Ctrl group; overall, there was a total of 55 proteins and peptides (Table 1). Information on the protein interactors identified with high accuracy in each Co-IP are reported in Supplementary Tables S2–S6; they include UniProt-KB codes, gene symbols, molecular weights, number of amino acids, score, coverage (%), number of unique peptides identified by Proteome Discoverer (Pd) software, and eventual post-translational modifications (PTMs).

#### 2.3.1. Proteins Identified in Co-IPs from Ctrl and Patient Samples

Eight proteins, cystatin D-C_26_ comprised, have been found to always be present in Co-IPs from both healthy Ctrls and patients (Table 1), probably most represented among the 55 identified interactors. We considered the C_26_ variant of cystatin D to be identified even if the tryptic fragment containing the cysteine residue at 26 position was not detected in our samples, since a specific Ab for the C_26_ variant was used for the co-immunoprecipitation. However, our results did not exclude that the R_26_ variant could also be part of the immunoprecipitated mPC. In addition, malignant brain tumors 1 protein (DMBT1), desmoplakin, mucin-5B, polymeric immunoglobulin receptor (PIgR), S100A9, and serpin B3 have also been detected in all the samples, as well as the form without the M_1_ residue of fructose-bisphosphate aldolase A.

14-3-3 protein sigma, which is the N-terminal acetylated form of ADP/ATP translocase 2 after M_1_ removal, the N-terminal acetylated cystatin B, and lactotransferrin were all identified in both Ctrl and SM groups. Cystatins S and SN, cornifelin, and prolactin-inducible protein (PIP) were identified in Ctrls and SM+C and SM−C subgroups.

Since these components were commonly detected in both healthy controls and patients, we named them “basal mPC components”.

#### 2.3.2. Proteins Identified in Co-IP from Ctrl Samples

Nine proteins were identified only in healthy Ctrl group (Table 1); among them, the N-terminal acetylated 14-3-3 protein zeta/delta, actin-related protein 2/3 complex subunit 4 (ARPC4), cathepsin G, cofilin-1, kallikrein-10, lysozyme C, and prohibitin.

#### 2.3.3. Proteins Identified Only in Co-IPs from Patient Samples

Several proteins and peptides have been detected specifically in the patients: leukocyte elastase inhibitor (also named serpin B1) and phosphoglycerate kinase 1 were identified in all patients (Table 1). Basic salivary proline-rich protein 2 (bPRP-2L) and submaxillary gland androgen-regulated protein 3B (P-B peptide) were identified in both SM+C and SM−C subgroups, but not in the entire SM group. bPRP-2L and P-B peptide were probably not present in the cystatin D-C_26_-linked mPC from each patient, and thus they were defined as under-represented components difficult to detect in the entire SM group. Nine proteins were identified in the Co-IP from the entire SM patient group but not in the patient subgroups (Table 1), among them azurocidin, the N-terminal acetylated form, after M_1_ removal, of fatty acid-binding protein 5 (FABP-5), glutamine synthetase, junction plakoglobin, neutrophil defensin 1, plakophilin-1, and S100A8.

In some cases, the sequence identified by the Pd software could not be attributed to a certain naturally occurring peptide. For example, the code of neutrophil defensin 1 (P59665) identifies both α-defensin 1 and α-defensin 2, which share the sequence except for the A_1_ residue present in α-defensin 1 and removed in α-defensin 2. Our (HR)-MS/MS analysis did not distinguish the two peptides, due to the failure to detect the N-terminal tryptic fragment. In the case of bPRP-2L, the code P02812 identifies several mature bPRPs, typical salivary peptides originating from pro-protein bPRP-2L [25], which are IB-1, P-J, P-F peptides, and IB8a protein. Indeed, the (HR)-MS/MS analyses individuated a fragment corresponding to a repeat sequence of 37 amino acid residues shared among these peptides. In addition, the fragment 1-57 belonging to either P-J peptide or IB8a protein was identified. However, it was, also possible to characterize sequences specific to certain bPRPs, such as the fragment 100-119 of IB8a carrying the serine residue at 100 position and thus identifying specifically the variant CON1+ of this protein that can undergo glycosylation [25]. Moreover, the fragment 1-57 of P-F peptide and the entire P-H peptide have been identified.

Some proteins/peptides were found specifically associated with either mastocytosis with systemic and cutaneous symptoms or with mastocytosis with only systemic symptoms (Table 1). Cystatin D-C_26_ interactors characterizing SM+C samples were as follows: annexin A1, caspase-14, filaggrin, hornerin, protein disulfide-isomerase (PDI), tubulin beta-4B chain, and zinc-alpha-2-glycoprotein. The proteoform of annexin A1 carrying an N-terminal acetylation after M_1_ removal and with a phosphorylation at the S_37_ was identified.

Cystatin D-C_26_ interactors characterizing SM−C samples were as follows: annexin A3, transketolase, dermcidin, glyceraldehyde-3-phosphate dehydrogenase (GAPDH), histatin-1 (Hst-1), suprabasin, tropomyosin beta chain, vimentin, and the N-terminally acetylated forms, after M_1_ removal of F-actin-capping protein subunit alpha-1 and L-lactate dehydrogenase A chain (LDH). It was not possible to identify which proteoform, phosphorylated or non-phosphorylated, of Hst-1 was present in the mPC.

### 2.4. Biological and Functional Analysis

The proteins/peptides identified as interactors of cystatin D-C_26_ in Ctrls, SM+C, and SM−C patient subgroups were submitted to biological pathway analysis. The results are reported in Table 2, Table 3 and Table 4, which show the data obtained using enrichment analyses performed against both the GO and Reactome databases. These two types of analysis almost always provided overlapping results, with the exception of some biological processes individuated by only one of the two databases. The biological processes associated with proteins commonly detected in all the groups are highlighted in light blue. The basal mPC components, detected in both Ctrls and patient groups, were found to be involved principally in three biological pathways (Table 2): (a) the sensory perception, as cystatins S, SN, and D, lactotransferrin, and 14-3-3 protein sigma; (b) the same proteins, together with serpin B3, S100A9, PIP, PIgR, and cystatin B, resulted in participation with PPIs implicated in the modulation of endoprotease activities; and (c) S100A9 and lactotransferrin were found to also be involved in the antimicrobial protein pathway.

The cystatin D-C_26_ partners in Ctrls (Table 2) are peculiarly implicated in tissue homeostasis, as are cystatin S, lactotransferrin, lysozyme C, fructose-bisphosphate aldolase A, PIP, PiGR, and 14-3-3 protein sigma. The antimicrobial protein pathway in Ctrls included lysozyme and cathepsin G, which were never detected in the patients, and DMBT1, in addition to S100A9 and lactotransferrin. Moreover, these antimicrobial proteins were found to be involved in immune response, innate system, and neutrophil degranulation pathways, together with serpin B3, cystatin B, PIgR, ARPC4, cofilin 1, mucin 5B, fructose-bisphosphate aldolase A, 14-3-3 protein zeta/delta, and desmoplakin.

Specific biological processes highlighted in SM−C patients were the glucose metabolism (phosphoglycerate kinase 1, GAPDH, LDH, and fructose-bisphosphate aldolase A), and the protein nitrosylation (S100A9 and GAPDH) (Table 3). The antimicrobial response included several pathways identified with different IDs and involving different protein networks than those identified in the Ctrl and SM+C patients (Table 3). These networks contained Hst1, GAPDH, mucin 7, dermicidin, vimentin, and annexin A3, antimicrobial peptides/proteins mediating the humoral immune response, specifically detected as cystatin D-C_26_ partners in SM-C patients. The same components were individuated as part of the network involved in the modulation of the interactions with symbiont microbiota, as well as in the pathways related to the innate immune system and neutrophil degranulation (Table 3), where F-actin capping protein subunit alpha-1 has also been identified.

The enrichment analysis individuated biological pathways specific to the SM+C patients, related to the epithelial and keratinocyte differentiation (Table 4). The protein networks involved in these processes comprised some cystatin D-C_26_ partners identified only in SM+C patients, as filaggrin, hornerin, annexin A1, and caspase 14, in addition to desmoplakin, cornifelin, DMBT1, which are basal mPC components, and phosphoglycerate kinase 1, which has been found in all the patient groups. Biological pathways similar to those individuated in the other groups involved different protein networks where components specific to the SM+C patients were identified (Table 4). Annexin A1, for example, was included in the regulation of hydrolase activity, as well as in the immune response and the neutrophil degranulation included together with tubulin beta-4B chain, hornerin, and protein disulfide-isomerase; finally, zinc-alpha-2-glycoprotein was included in the network implicated in the sensory perception process.

Supplementary Table S7 reports the biological processes and cellular localization, known for each of the peptides/proteins listed in Table 1, as provided by Pd software based on the GO term databases. 

## 3. Discussion

Changes of the salivary protein profile as well as altered protein–protein interactions can reflect a systemic pathological condition [10,11,12] and can be suggestive for a mechanism of disease pathogenesis and for individuation of novel therapeutic targets. Considering the difficulties in classifying the different forms of mastocytosis, and often in diagnosing indolent forms of the disease, all the efforts to discover new characterizing molecular signals useful for individuating potential diagnostic biomarkers and new therapeutic targets are of great interest for the scientific community engaged on the topic of mastocytosis. From this point of view, investigations based on proteomic platforms and mass spectrometry (MS) analysis of protein profiles are certainly able to provide results with high throughput, accuracy, and sensitivity. The limited number of proteomic studies performed in the field of mastocytosis individuated pro-inflammatory factors as novel potential blood biomarkers for systemic mastocytosis (SM) [26] and proinflammatory mechanisms possibly involved in pathogenesis of mastocytosis, as the upregulation of the signaling pathways downstream of Toll-like receptor 4, TNF-a, and IFN-g [27]. An interesting approach coupling plasma proteomics with single-cell transcriptomic analysis in SM patients and healthy subjects identified some cytokines and interleukins as specific biomarkers [28]. In a previous proteomics study, performed using a top-down platform, we highlighted the presence in saliva of peripheral and non-invasive candidate biomarkers of mastocytosis, some of them able to distinguish subtypes of SM with and without cutaneous symptoms (SM+C and SM−C), such as the R_26_ variant of cystatin D [13]. The present exploratory study, focused on the C_26_ variant of cystatin D, which is able to participate with multiprotein complexes’ (mPCs) formation [21] and it is not detectable with a top-down proteomic approach, demonstrated not only that salivary cystatin D-C_26_ interactome exhibited peculiar protein composition in healthy subjects (Ctrls) and in patients affected by mastocytosis, but also the different protein composition between the two subgroups of patients affected by systemic mastocytosis with and without cutaneous symptoms. Furthermore, although most of the biological processes involving the cystatin D-C_26_ interactors, identified by enrichment analyses, were common to both the Ctrl and patient groups, it was possible to reveal some biological processes peculiar to the Ctrl group or SM+C or SM−C patient subgroup. Thus, the different composition of the cystatin D-C_26_-bound mPCs was reflected in diverse PPIs in which the interactors were implicated.

### 3.1. Limitations of the Study

In the present study, we intended to examine the salivary cystatin D-C_26_ interactome in mastocytosis patients and healthy individuals through a qualitative, and not-quantitative, analysis. The restricted number of samples available for the SM−C group, which is a rare form of systemic mastocytosis, and the low volumes of whole saliva collected from several individuals, obliged us to prepare pooled samples and to develop an experimental design with the aim of defining only qualitative changes. Despite the limitations, very promising results were obtained, which inspired a successive investigation with the aim of evaluating quantitative changes by applying our AP-MS method to single samples and to validate the MS data using immunodetection techniques. Another limitation of the present study was the lack of whole saliva samples collected from patients with cutaneous mastocytosis (CM). It was not possible to collect such samples for this investigation, due to the very low number of available CM patients, and thus it was not possible to characterize the composition of the salivary D-C_26_-bound mPC in CM patients. This would have allowed the identification of interactors and PPI networks specifically related to skin disorders of mastocytosis also in SM+C patients; however, this remains an objective to be pursued in the future.

### 3.2. Cystatin D-C_26_ Interactors and PPI Common to Ctrls and Patients

The basal mPC components, identified in both Ctrls and patients, appeared to constitute a “scaffold” of multifunctional proteins mainly involved in innate and acquired immunity of the oral cavity, such as PIP, PIgR, S100A9, mucin 5B, lactotrasferrin, cystatins D-C_26_, B, S, and SN, and DMBT1. They are included among the “salivary defense proteins” with overlapping actions able to act synergistically and/or additively in a “multi hit” network type approach [16]. These proteins play antibacterial and antiviral activities, bind bacterial lipopolysaccharide (LPS), which is responsible for tissue-destructive inflammatory response and in some cases they exhibit immunomodulatory properties and the ability to regulate inflammatory processes such as cystatins B, S, SN, DMBT1, and S100A9, as well as the ability to bind to each other as mucin 5B and DMBT1 [15,16]. Accordingly, our biological pathway analysis based on the Reactome database individuated a common antimicrobial pathway involving S100A9 and lactotransferrin in both healthy Ctrls and patients. Some basal mPC components were found to participate in the sensory perception network, such as cystatins S, SN, and D-C_26_, lactotransferrin, and 14-3-3 protein sigma. This last protein appeared particularly interesting, since it can regulate a large spectrum of general and specialized signaling pathways binding many partners, usually by recognition of a phosphoserine or phosphothreonine motif [29]. It is, therefore, not usual to have identified this protein in our samples. Several basal mPC components were found to be part of pathways implicated in the regulation of endoprotease activities, among them cystatins and serpin B3, which are cysteine protease inhibitors acting mainly on cathepsins with protective effects. Cystatins S, SN, and D are typically secreted in the oral cavity by the salivary submandibular and sublingual glands, although they can also be detected in other biological fluids and organs [15,20]. Cystatin B shows a broader tissue expression [15]. Cystatin B and D may have nuclear effects protecting transcription factors from the proteolytic activity of cathepsin L [15]. Cystatin D exhibits cell antimigratory and antiproliferative effects probably due also to a cathepsin-independent mechanism [30]. It is considered to be a tumor suppressor, since it may decrease the proliferation, migration, and invasion of colon carcinoma cells [30] probably due to its ability to control the transcription of specific genes involved in the modulation of the cell junction, actin cytoskeleton, and cell adhesion [30]. Furthermore, cystatin D is able to change the pattern of cytokine secretion, such as fibroblast growth factor 4, CX3CL1/fractalkine, neurotrophin 4, and others. The ability of cystatin D to regulate cytokine expression and secretion could be of great interest in the field of mastocytosis research, since several cytokines have been suggested as candidate diagnostic biomarkers and therapeutic targets [26,28]. Serpin B3 is a papain-like cysteine protease inhibitor expressed in the normal squamous epithelium in several organs, it is prevalently cytosolic even if may be secreted in serum [31]. It is physiologically involved in the regulation of the squamous epithelium differentiation and overexpressed in neoplastic tissue of epithelial origin, where it is able to modulate the host immune response against tumor cells. Serpin B3 may protect from bacterial, viral cysteine proteases, and mast cell chymase [31].

### 3.3. Cystatin D-C_26_ Interactors and PPIs Peculiar to Ctrls

Several cystatin D-C_26_ interactors were detected in the mPC from healthy subjects but not in patient samples. The prevalent biological pathways involving these cystatin D-C_26_ interactors include basal tissue homeostatic processes, and the related protein networks comprised lysozyme C, an antibacterial small protein participating in natural oral defense [16], in addition to several basal mPC components. Some antimicrobial pathway associated networks appeared to be specific to the mPC characterized in the Ctrl group, since they included lysozyme C and cathepsin G, not identified in the patient samples. Cathepsin G is an interesting multifunctional serine protease considered a crucial protein in the maintenance of the delicate balance between tissue protection and destruction during inflammation [32]. The secreted form is released by neutrophils and mast cells during the immune and/or inflammatory response; it stimulates production of cytokines and chemokines responsible for the activation and mobilization of immune cells to the site of pathogen and/or tissue damage [32]. The cellular form is also synthesized in non-myeloid cells and it was suggested that it may be involved also in normal physiological processes such as digestion, smooth muscle contraction, epithelial repair, and tissue remodeling [32]. Moreover, the antimicrobial protein networks included DMBT1, a basal mPC component also named salivary agglutinin and implicated in mucosal defense system, immune defense, binding with LPS, and in epithelial differentiation [16]. DMBT1 can bind a large range of oral pathogens and regulate either bacterial/viral invasion and/or surface exclusion by promoting their adhesion on oral surfaces [33].

The biological pathways related to the immune response, innate immune system, and neutrophil degranulation were associated with specific protein networks in the Ctrl group. These protein networks included some cystatin D-C_26_ interactors detected only in Ctrl group, such as cathepsin G, lysozyme C, ARPC4, cofilin 1, and 14-3-3 protein zeta/delta, in addition to several basal mPC components. 14-3-3 protein zeta/delta, similarly to the 14-3-3 protein sigma, is a protein usually participating in the formation of multiprotein complexes [29]. ARPC4 and cofilin 1 are important modulators of the actin-polymerization in the cytoskeleton [34]; therefore, they represent crucial regulators of the development, adhesion and migration of the immune cells, which are processes requiring cytoskeleton remodeling. For this reason, ARPC4 and cofilin 1 are part of the essential components of the so-called homeostasis-altering molecular processes that induce the activation of innate immune signaling pathways [34].

It is interesting to highlight that ARPC4, cathepsin G, cystatins B and D, mucin 5B, PIP, and S100A9 were also characterized in another salivary interactome that was associated with cystatin B. It was previously investigated in salivary samples from healthy subjects and patients affected by Alzheimer’s disease [35]. Their constant presence in these multifunctional multiprotein complexes suggests their basal role in the protein defense network of the mouth.

### 3.4. Cystatin D-C_26_ Interactors Peculiar to Patients

Several cystatin D-C_26_ interactors identified in this study were revealed only in samples from patients, suggesting the existence of specific PPIs associated with the pathological condition. Some of these proteins were characterized in all groups, SM, SM+C, and SM−C, some only in both SM+C and SM−C subgroups, and others only in the entire SM group. Some peptides secreted by salivary glands [36] were detected, such as bPRPs derived from the pre-secretory maturation of the bPRP-2 pro-protein, which may be involved in the constitution of the protein pellicle covering oral surface and in the complexing of dietary tannins [37,38]; P-B peptide originated from maturation of the submaxillary gland androgen regulated protein 3B (SMR3B). Our enrichment analysis included P-B peptide as part of the protein networks associated with the regulation of endopeptidase and hydrolase activity in both the SM−C and SM+C subgroups. The potential implication in the mastocytosis of bPRPs and P-B peptide remains a puzzling point. Although it is not possible to make quantitative considerations in this study, it should be emphasized that P-B peptide was found at a significantly lower concentration in SM than in Ctrl subjects in our previous proteomic study, while the salivary naturally occurring P-B fragments were suggested as potential markers to differentiate SM−C and SM+C patients [13].

Leukocyte elastase inhibitor, also named serpin B1, is a neutrophil serine protease inhibitor involved in the regulation of the innate immune response, inflammation, and cellular homeostasis [39]. It primarily protects cells from proteases released by neutrophils and activated mast cells during stress or infection, such as neutrophil proteases elastase, cathepsin G, chymase, and others [39,40,41,42]. It is interesting to underline that while serpin B1 was detected in the mPC characterized in the patients, suggesting a protective role of this protein in our patients and probably a response to the mast cell activation, cathepsin G was instead found to be a cystatin D-C_26_ interactor specific to the healthy controls, suggesting a basal homeostatic role of this protease in the oral cavity.

Some interactors characterized in patients are proteins released from neutrophils and involved in the immune and inflammatory responses, such as the neutrophil defensin 1, azurocidin, and S100A8. α-defensin 1 and its proteolytic derivative α-defensin 3, comprising the “Neutrophil defensin 1” term, are broad antibacterial and antiviral peptides, also able to contribute to immune modulatory activity and to promote the cytokines’ induction [16]. Azurocidin is an antibacterial glycoprotein with strong monocyte- and fibroblast-specific chemotactic action, which recognizes Gram-negative bacteria through its strong affinity to LPSs [43]. S100A8, together with S100A9, represent the main protein content of the neutrophils [44]. S100A8 may interact with Toll-like receptors (TLRs), activating the innate immune system and mediating inflammation through induction of cytokine secretion and affecting monocyte and macrophage behavior [44]. S100A8 exerts anti-inflammatory activity when modified by nitrosylation of its cysteine residue and can act as an ROS/RNS scavenger against oxidative stress [45]. Remarkably, S100A8 was found to be more concentrated in SM patients than in healthy controls in our previous investigation [13], and thus, its detection in the mPC associated with cystatin D-C_26_ in the SM group encourages us to carry out further investigations on the possible use of this protein as a candidate SM biomarker. Furthermore, glutamine synthetase was particularly interesting for its regulatory role in nitrogen metabolism; it decreases free ammonia cellular levels by incorporating it into glutamine and so reducing its toxicity. This is a function that makes this enzyme crucial for the synaptic neurotransmission glutamate-dependent and for the protection of the brain by ammonia toxicity [46]. It is strongly expressed in cells of the tumor microenvironment, such as fibroblasts, adipocytes, and immune cells, in which it is responsible for the acquisition of protumoral phenotypes. However, it should be underlined that the metabolic role of glutamine synthetase within the tumor is complicated by different factors, such as the type of tissue/cell, the metabolic heterogeneity of the different regions of a tumor, many transcriptional factors, and oncogenes, all factors that overall produce metabolically divergent patterns. Nevertheless, the presence of glutamine synthetase typically in SM samples may be correlated with the neoplastic feature of activated mast cells in the disease and thus with their metabolic reprogramming. In addition, recent insights demonstrated its role in modulating immune function [47]. Indeed, glutamine synthetase, being sensitive to redox balance, can be oxidized with consequent loss of function that in microglia triggers a strong inflammatory response to LPS producing many inflammatory mediators and effectors. The redox sensitivity of S100A8 and glutamine synthetase might represent a strategy by which several mechanisms relevant to the inflammatory response are modulated, and it might also be of interest in the mastocytosis pathogenesis. Indeed, mastocytosis has been associated with a state of increased oxidative stress [48].

Another protein with antibacterial activity detected in the mPC characterized in SM patients was FABP5, a cytosolic carrier for long-chain fatty acids and active lipids, such as endocannabinoids, which is implicated in the LPS-induced cytokine production of mast cells [49].

#### 3.4.1. Cystatin D-C26 Interactors and PPIs Peculiar to SM+C Patients

In SM+C patients, cystatin D-C_26_ interactors resulted predominantly and specifically implicated in epithelial and keratinocyte differentiation. The associated networks included some proteins detected only in SM+C group, such as filaggrin, caspase-14, hornerin, and annexin A1, together with basal mPC components such as desmoplakin, cornifelin, and DMBT1, and the phosphoglycerate kinase 1 commonly detected in patient but not in Ctrl samples. Filaggrin, hornerin, and caspase-14 are components of the epidermal cornified cell envelopes, involved in processes of regulation and homeostasis maintenance of the epidermal barrier and in its protection from water loss [50,51]. Filaggrin is the main substrate of caspase-14, which is a peculiar caspase mostly expressed in the cornifying epithelia, where is implicated in the programmed cell death/cornification process by catalyzing the proteolysis of the profilaggrin into filaggrin and the filaggrin into free amino acids [52]. Filaggrin in turn is essential for the aggregation of cytoskeletal keratin filaments. Desmoplakin is a multifunctional protein constituting desmosomes and is thus crucial for preserving tissue integrity and modulating cell behavior; indeed, it exhibits viral receptor activity and is implicated in cell adhesion and cell motility [53]. DMBT1 may participate in remodeling the actin cytoskeleton during exocytosis [54]. Mast cells may have a protective role against chronic inflammatory skin diseases condition via the regulation of epidermal barrier function and the promotion of skin homeostasis [51]; alterations of these functions are typical of systemic mastocytosis with cutaneous signs and the fact that this state may be reflected in the oral cavity, as our findings suggested, could be useful in the perspective to identify specific peripheral biomarkers for this form of the disease.

The participation of phosphoglycerate kinase 1 in this network was intriguing; indeed, apart from the canonical metabolic role, it is involved in multiple biological processes, including angiogenesis, DNA replication and repair, the proliferation and metastasis of tumor cells, and cell invasion [55]. We might assume that the presence of phosphoglycerate kinase 1 only in patient samples and its inclusion in the PPI networks associated with the epithelial differentiation pathway may be linked to an altered cell proliferation process and to the neoplastic feature of mastocytosis. Interestingly, several proteins identified in SM+C group are mast cell activation factors, such as annexin A1, an anti-inflammatory factor secreted by mast cells, neutrophils, eosinophils, monocytes, epithelial, and T cells. It was demonstrated in a transgenic mouse model that the protein could regulate mast cell reactivity [56]. Similarly to phosphoglycerate kinase 1, annexin A1 can take part in a wide variety of cancer processes, including carcinogenesis, cell proliferation, invasion, apoptosis, and metastasis [57]. The enrichment analysis also included it in the regulation of hydrolase activity, as well as in immune response and neutrophil degranulation, together with protein disulfide-isomerase (PDI), tubulin beta-4B chain, and hornerin. PDI is abundantly secreted by mast cells during IgE and antigen activation. Moreover, it has been suggested that PDI activity is essential in the regulation of mast cell activation and that its inhibition may be beneficial for patients with allergic inflammation [58]. Tubulin beta-4B chain is the major constituent of microtubules in the cytoskeleton. In addition, this protein was indicated as a factor in regulating mast cell activation [59].

#### 3.4.2. Cystatin D-C26 Interactors and PPIs Peculiar to SM−C Patients

The cystatin D-C_26_-bound mPC in the SM−C patients was characterized by proteins implicated in the glucose metabolism network; some of these were detected only in SM−C group, such as GAPDH and LDH. In the same protein network, the enrichment analysis also identified phosphoglycerate kinase 1 and fructose-bisphosphate aldolase A, both not specific to the SM-C group. While on the one hand this result may suggest an alteration of glucose metabolic pathways, on the other it should be underlined that often these enzymes are moonlighting proteins with various interesting features and functional implications. LDH has been identified in SM patients as one of the prognostic factors for survival and considered useful to define criteria and subvariants for SM [60]. Elevated levels of LDH, in combination with other factors, are typical of aggressive SM (ASM) and MC leukemia (MCL) [60]. GAPDH exhibits immunomodulatory effects, demonstrated in vivo and in vitro during allergic responses [61]. Moreover, transketolase was also identified as a cystatin-D C_26_ interactor specifically in the SM−C group. It is an enzyme of the pentose phosphate pathway, and as already suggested for S100A8 and glutamine synthetase, it may be of interest if we consider that mastocytosis has been associated with a state of increased oxidative stress [48]. In support of this, our enrichment analysis identified GAPDH and S100A9 as part of the protein network implicated in the protein nitrosylation pathway in SM−C group. It was demonstrated that GAPDH and S100A9, as well as S100A8, are directly involved in S-nitrosylation and trans-nitrosylation of target proteins through the formation of S-nitrosylation complexes [62]. They are proteins propense to be nitrosylated at their cysteine residues (SNO) in oxidative stress conditions by either S-nitrosylases or non-enzymatic mechanism, in turn SNO-GAPDH and SNO-S100A9 can propagate the SNO modification on target proteins throughout the cell via non-enzymatic transnitrosylation. Moreover, GAPDH has been included in the protein networks associated with the antimicrobial and humoral immune response pathways, together with Hst1, mucin 7, dermicidin, annexin A3, and vimentin, specifically detected in SM−C group. The same components were individuated as part of the network involved in the modulation of the interactions with symbiont microbiota, as well as in the innate immune system and neutrophil degranulation pathways together with F-actin capping protein subunit alpha-1. Hst1 and mucin 7 are antimicrobial components typical of the salivary fluid [16], dermicidin is a peptide with a broad spectrum of anti-bacterial actions [63], while annexin A3 is restricted to neutrophils where it supports the microbicidal activity. Moreover, it was supposed that annexin A3 may promote neutrophil survival with its anti-apoptotic activity [64]. Vimentin and F-actin-capping protein subunit alpha-1, implicated in the organization of cytoskeleton, are indicated as factors able to regulate mast cell activation [59], as well as tropomyosin beta chain, another protein distinctive of the mPC characterized in SM−C group. Finally, suprabasin, an epithelial pro-inflammatory peptide, has also been indicated as possible regulator of mast cell activation via TLR4 leading to an increase in the inflammatory response, as demonstrated in psoriasis [65]. Since the salivary samples from SM−C patients used to prepare the pool intended for immunoprecipitation were fewer than those from SM+C patients it is interesting and remarkable to have found specific interactors associated with this rare form of mastocytosis.

Surprisingly, several proteins typically localized either in cytoplasm, mitochondria, or membranes were identified in the cystatin D-C_26_ interactome, they probably reach the oral cavity as a consequence of the turnover of mucosal and glandular epithelial cells, keratinocytes, or infiltrating leukocytes. What the role of these proteins may be in the salivary cystatin D-C_26_ interactome here characterized remains enigmatic; however, it appeared particularly amazing to have identified in the salivary cystatin D-C_26_ interactome proteins implicated in cell and tissue structural functions, some typically acting in the arrangement of the cytoskeleton and some composing desmosomal structures.

Indeed, it is stimulating to think that the detection of these proteins was not casual in the interactome under study but was related to its biological role in healthy and pathological conditions. To support this, it is worthy to emphasize that mast cell activation in allergic response and inflammation, mediated by the high-affinity receptor for IgE or other receptors, such as c-Kit and G protein-coupled receptors, is associated with changes of cell morphology, adhesion to substrate, exocytosis, and migration, processes for which the reorganization of cytoskeleton is a critical event [59]. Furthermore, several “structural” proteins here identified are indicated as factors able to regulate mast cell activation [59]. Additionally, it is stimulating to have found different protein compositions of the salivary cystatin D-C_26_ interactome in relation to the different conditions under study, healthy, systemic mastocytosis, and the presence or not of cutaneous symptoms in patients. Differences that were reflected on PPI networks and biological processes distinctive for the compared groups. These results supported the initial idea, which was to find, in the formation of salivary cystatin D-C_26_-bound multiprotein complex, molecular traces of disease-related changes occurring in the biological pathways in which the cystatin D-C_26_ itself and its interactors are implicated. If in physiological conditions the cystatin D-C_26_ interactome seemed to play a biological role of protection, maintenance of homeostasis, and control of the innate immune system in the oral cavity, in the SM condition, on the other hand, molecular signs appeared potentially related to the activation of mast cells, to mechanisms of self-protection through the immune and/or inflammatory response, and thus to PPI alterations that could reflect disease pathogenesis. Although preliminary, these results appeared promising for future studies. Indeed, we considered this exploratory AP-MS study a starting point for further in-depth investigations designed to examine quantitative changes in the cystatin D-C_26_ interactome on a larger cohort of patients, on samples analyzed individually and not in a pool and with a comparison with other immunological diseases, with the aim of discovering novel candidate salivary biomarkers of systemic mastocytosis.

## 4. Materials and Methods

### 4.1. Reagents and Instruments

Protein A/G PLUS-Agarose, mouse monoclonal Ab against cystatin D-C_26_, HRP conjugated anti-mouse secondary Ab, and nonspecific primary Ab were purchased from Santa Cruz Biotechnology (Dallas, TX, USA). Chemicals and reagent for SDS-PAGE and Western blot were purchased from Bio-Rad (Hercules, CA, USA), such as the ChemiDoc MP Imaging System. All the chemicals and reagents used for in-gel tryptic digestion and mass spectrometry analyses and the kit for the bicinchoninic acid (BCA) assay were purchased from MERCK-Sigma-Aldrich (Darmastadt, Germany), such as the NanoDrop 2000 Spectrophotometer used for determination of the protein concentration. HPLC-high-resolution ESI-MS and MS/MS experiments were carried out using an Ultimate 3000 nano-HPLC apparatus (Dionex, Sunnyvale, CA, USA) coupled through an EASY-spray nano ESI source (Thermo Fisher Scientific, San Jose, CA, USA) the LTQ-Orbitrap Elite mass spectrometer. The trap column (5 mm × 300 µm I.d.) and the EASY-spray^TM^ C18 nanoHPLC column (150 mm × 50 µm, particle size of 2 µm) were purchased from Thermo Fisher Scientific.

### 4.2. Study Subjects and Controls

A total of 32 patients with systemic mastocytosis (SM, 17 males and 15 females, mean age ± SD: 48 ± 12), were enrolled from the Internal Medicine and Immunology outpatient’s clinic of the University of Cagliari. A total of 28 out of the 32 patients manifested both cutaneous and systemic symptoms (SM+C, 13 males and 15 females, mean age ± SD: 48 ± 13), whereas the remaining 4 only manifested systemic symptoms (SM−C, 4 males, mean age ± SD: 47.5 ± 10). A total of 20 sex/age matched healthy Ctrls (11 males and 9 females, mean age ± SD: 39 ± 10) were enrolled as volunteers among the staff of the Department of Life and Environmental Science, University of Cagliari. Written informed consent was obtained from each subject involved in the study; the informed consent process agreed with the latest stipulations established by the Declaration of Helsinki. Local review boards approved the study, and in view of its observational nature a formal ethical committee approval was obtained (Prot.PG/2020/9425). Demographic and clinical features of the included patients are reported in Table 5. Diagnosis was made according to the diagnostic criteria provided by WHO in 2016 considering clinical assessments, tryptase levels in blood, and in several case the genetic analysis [2]. A total of 18 SM patients carried out the mutation D816V on *c-Kit* gene, while only a patient (#9) exhibited M541L mutation. All patients suffered from gastrointestinal symptoms, while 34.4% of patients manifested oral aphtosis, of which 1 SM−C and 10 SM+C.

### 4.3. Sample Collection

Patients and Ctrls included in this AP-MS study were the same as those included in our previous study focused on top-down salivary proteomics [13]. The samples devoted for the two studies were collected from the same subjects at the same collection time. All the samples of unstimulated whole saliva were collected during the morning (between 10:00 a.m. and 12:00 p.m.). Donors, in fasting conditions, were invited to sit assuming a relaxed position and to swallow. Whole saliva was collected as it flowed into the anterior floor of the mouth with a soft plastic aspirator for less than 1 min, and was transferred to a plastic tube and cooled on ice. Whole salivary samples intended for the AP-MS investigation were immediately diluted in a 1:1 *v*/*v* ratio PBS buffer (270 mM NaCl, 5 mM KCl, 20 mM Na_2_HPO_4_, 4 mM KH_2_PO_4_) containing a cocktail of protease inhibitors (mini-cOMPLETE EDTA-free in 1:2.5 *v*/*v* ratio with PBS buffer–Roche Diagnostics GmbH, Darmstadt, Germany), and stored at −80 °C until the Co-IP experiments, performed 3 months after the sample collection. Total protein concentration (TPC) was determined by BCA assay according to manufacturer instructions.

### 4.4. Immunodetection of Cystatin D-C_26_

The immunodetection of cystatin D-C_26_ was performed using Western blot analysis, firstly on nine single whole salivary samples from Ctrls as exploratory experiment to optimize the immunodetection conditions. Then, the method was applied to the co-immunoprecipitated (Co-IP) samples as described in the Section 4.5. For Western blot and immunodetection analysis the mouse monoclonal Ab against the variant cystatin D-C_26_ and 6µg of total proteins for each sample were used. SDS-PAGE was performed under non-reducing (NR) and reducing (R) conditions using 4–15% T mini precast gels (Bio-Rad), a voltage of 180V and the MW standard Precision Plus Protein^TM^ WesternC^TM^ Blotting Standard (Bio-Rad). After SDS-PAGE separation proteins were transferred to a 0.2 µm polyvinylidene difluoride (PVDF) membrane according to the instructions provided with the Trans-Blot Turbo system (Bio-Rad). After the transfer, PDVF membranes were immersed in blocking solution (5% BSA in Tris-buffered saline containing 0.05% tween-20, TBS-T) under stirring for 1 h. Then, the membranes were incubated for 1 h with the mouse monoclonal primary cystatin D-C_26_ Ab (1:1000, Ab:TBS-T), and for 1 h with the HRP conjugated anti-mouse secondary Ab (1:50,000 Ab:TBS-T). Finally, they were immersed for 5 min in Bio-Rad ECL Western Clarity detection solution, and the images were acquired using ChemiDoc MP Imaging and analyzed with Image Lab 4.0.1.

### 4.5. Co-Immunoprecipitation Experiments and SDS-PAGE

Salivary pools from SM−C and Ctrl groups were used for the co-immunoprecipitation procedure; we used different volumes from each sample based on their TPC to prepare the pools, so that each sample contributed with the same protein amount. For SM and SM + C groups, two biological replicates were prepared utilizing salivary whole samples from different patients, they were composed as it follows: SM1, 16 samples, 8 males, 8 females, 48 ± 12 yr, 2 SM−C, and 14 SM+C samples; SM2, 16 samples, 9 males, 7 females, 47 ± 13, 2 SM−C, and 14 SM+C samples; SM+C1, 14 samples, 6 males, 8 females, and 48 ± 12 yr; and SM+C2, 14 samples, 7 males, 7 females, and 47 ± 13 yr. In addition, a second salivary pool from Ctrl group, named NEG (11 males and 9 females, 39 ± 10 yr), was prepared for identification of unspecific interactors. Also, in this case, different volumes were used to prepare the pools from each sample based on their TPC, so that each sample contributed with the same amount of protein. The same total protein amount, 400μ g, from each pool were treated with preclear solution containing a nonspecific primary Ab and the Protein A/G PLUS-Agarose (Santa Cruz Biotechnology), following manufactory instructions, to reduce non-specific binding to immunoglobulins, and especially to remove mouse monoclonal Abs from IgG subclasses. The pools were then incubated with the specific primary cystatin D-C_26_ Ab except for the NEG pool that was incubated with a nonspecific primary Ab, under stirring over night at 4 °C. Cracking solution (0.125 M Tris/HCl pH 6.8, 4% SDS) was used to break the interaction between AG protein and the Ab and between Ab and proteins. After boiling and centrifugation, the supernatant was recovered and its TPC measured by BCA assay with the NanoDrop 2000 Spectrophotometer. A total of 4 μg proteins of each Co-IP were then separated by SDS-PAGE under NR and R conditions using the protocol previously indicated. For NR Co-IPs, the SDS-PAGE separation was only intended for the Western blot analysis of the Co-IP samples with cystatin D-C_26_ Ab, applying the experimental conditions described above. For R Co-IPs, the SDS-PAGE separation was made in double, one addressed towards the Western blot analysis with cystatin D-C_26_ Ab and the second one stained with Bio-Safe^TM^ Coomassie G250 and used for the in-gel tryptic digestion followed by high-resolution MS/MS analysis. In this second SDS-PAGE separation to allow the loading of the samples in duplicate, two gels were contemporaneously prepared (Supplementary Figure S2a,b). After tryptic digestion, fragment peptides from the corresponding slices of the same duplicated Co-IP were unified. In each of these gels the Ctrl Co-IP was loaded in double wells with the same protein amount, but only two of these four lanes were used for the bottom-up analysis, and they are indicated in panel (a) of Figure S2 with asterisks.

### 4.6. Tryptic Digestion and High-Resolution MS/MS Analysis

The slices were excised from the gel as shown in Figure S2a,b and transferred to fresh tubes; for de-staining and peptide extraction procedures, the protocol of Gundry et al. was applied [66]. Extraction was repeated twice and followed by drying under vacuum and resuspension of the tryptic peptides in 0.1% formic acid (FA) for the nanoHPLC-ESI-high-resolution MS/MS analysis by an LTQ-Orbitrap Elite apparatus or stored at −80 °C in dried form until the analysis. Tryptic peptides from corresponding slices belonging to the same Co-IP, loaded in duplicate into the gel (Figure S2a,b), were unified before (HR)-MS/MS analysis. In this way, we analyzed separately six tryptic samples for each of the seven Co-IPs, Ctrl, NEG, SM−C, SM+C1, SM+C2, SM1, and SM2. The two biological replicates of samples from the entire patient group (SM) and from the subgroup of patients with both systemic and cutaneous symptoms (SM+C) were separately analyzed by (HR)-MS/MS.

Elution solvents were as follows: A, 0.1% FA in water and B, 0.1% FA in ACN–water 80/20 (*v*/*v*). The flow was 300 nL/min. Samples were injected at 10 μL/min in 96% solvent A (0.1% FA). The used gradient was as follows: 0–3 min, 96% solvent A; 3–70 min, 4–50% solvent B; 70–90 min, 50–80% solvent B; 90–92 min, 90% solvent B; 90–100 min, 90% solvent B; and 101–120 min, 96% solvent A. Full MS experiments were performed in positive ion mode with a mass range from 350 to 1600 *m*/*z* at a resolution of 120,000. The capillary temperature was 275 °C, the source voltage was 1.7 kV. In data-dependent acquisition mode, the ten most abundant ions were acquired and fragmented by using collision-induced dissociation (CID) with a 35% normalized collision energy for 10 ms, isolation width of 5 *m*/*z*, and activation q of 0.25. Spectra were acquired by Xcalibur software (version 3.0, Thermo-Fisher Scientific, Waltham, MA, USA) and analyzed using the Proteome Discoverer (PD) software (version 2.5, Thermo Fisher Scientific), with the search engines MS Amanda (version 2.0, University of Applied Sciences Upper Austria, Research Institute of Molecular Pathology) and SEQUEST HT (University of Washington, in license to Thermo Electron Corp., San Jose, CA, USA). Proteome Discoverer (Pd) software used the Uniprot-KB human protein sequence database (20375 entries, release 2022_01). To obtain the identification of all the proteins/peptides present in that Co-IP, MS data from gel slices of the same Co-IP (SM1, SM2, SM+C1, SM+C2, SM−C, Ctrls, or NEG) were merged in the PD analyses with the following parameters: up to two missed tryptic cleavages; peptide mass tolerance 10 ppm and fragment ion mass tolerance 0.6 Da; and FDR were: 0.01 (strict) and 0.05 (relaxed). Peptides were filtered for high confidence and a minimum length of 6 amino acids, proteins were filtered for a minimum of 2 unique peptides. Contaminants from sample manipulation (keratins) were excluded, as well as non-specifically bead-binding proteins (hemoglobin, histones, and immunoglobulins) [23,24]. The considered post-translational modifications were: carbamidomethylation of cysteine (fixed), oxidation of methionine, serine/threonine/tyrosine phosphorylation, C-terminal pyroglutamate, and N-terminal acetylation (dynamic). For the label-free quantification (LFQ), the MS data from all the different Co-IPs (SM1, SM2, SM+C1, SM+C2, SM−C, Ctrls, and NEG) were loaded in a unique comparison analysis in the Pd software keeping the parameters above illustrated. In this way, we obtained an accurate and comparable determination of the LFQ abundances of the proteins/peptides identified as interactors of cystatin D-C_26_ in the Co-IPs from the four groups. The protein abundances were normalized against the total peptide amount in the precursor ion quantifier node of Pd software, and statistically analyzed as described in the next paragraph with the precise aim of excluding unspecific interactors. The MS data have been deposited to the ProteomeXchange Consortium (http://ww.ebi.ac.uk/pride (accessed on 5 November 2019)) via the PRIDE, version 2.5.2 [67], partner repository with the dataset identifier PXD042567.

### 4.7. Statistical Analysis

The statistical comparison among the quantitative MS data determined in the patient and Ctrl groups was performed against the NEG group with Perseus tool (version 2.0.3, Max-Planck-Institute of Biochemistry) following the instructions provided for label-free interaction data [68]. In the case of the two Co-IPs obtained from SM salivary pools (SM1 and SM2), and of the two Co-Ips obtained from SM+C salivary pools (SM+C1 and SM+C2), the mean of the LFQ abundances of every protein/peptide was calculated and used by Perseus software (version 1.6.15.0) during the data elaboration. LFQ intensities, provided by Pd software for SM, SM+C, SM−C, Ctrl and NEG Co-IPs, were loaded as data matrices into Perseus and transformed into Log2 scale. Student’s *t*-test was applied to perform the two-sample comparisons. Based on the fold-change threshold fixed at ±2.0 with 250 randomizations, in which the protein’s intensities were unchanged or higher in NEG, proteins were considered unspecific interactors and filtered out. Meanwhile, when a FC ≤ −2 was obtained with respect to NEG the protein/peptide was considered included among the cystatin D-C_26_ interactors.

### 4.8. Functional Enrichment Analysis

Enrichment and pathway analyses were performed using STRING v.11.5 [69] (latest access on July 2023) separately on the protein interactors specifically found in the salivary cystatin D-C26 interactome from Ctrl group (25 proteins), SM−C group (26 proteins), and SM+C group (22 proteins). The Gene Ontology (GO) biological process database (last update 05-2022) and Reactome pathway database (last update 05-2022) were tested for each group. The analyses were performed with a default medium confidence of 0.4 and FDR stringency at 5%. Active interaction sources were based on “experiments”, “co-expression”, and “co-occurrence”. FDR was corrected with Bonferroni step down. Information on biological function and cellular localization of each identified cystatin D-C_26_ interactors have also been provided by the analysis of Pd software based on the GO term databases.

## Figures and Tables

**Table 1:** Cystatin D-C_26_ interactors identified in salivary mPCs co-immunoprecipitated from Ctrl, SM+C, SM−C, and SM pooled samples. PTMs characterized are indicated, as well as the presence revealed in each group under study by the symbol “•”.

Uniprot-KB Code	Description	PTMs	Ctrl	SM+C	SM−C	SM
P28325	Cystatin D-C_26_		•	•	•	•
Q9UGM3	Deleted in malignant brain tumors 1 protein (DMBT1)		•	•	•	•
P15924	Desmoplakin		•	•	•	•
P04075	Fructose-bisphosphate aldolase A	All: M_1_-loss	•	•	•	•
Q9HC84	Mucin 5B		•	•	•	•
P01833	Polymeric immunoglobulin receptor (PIgR)		•	•	•	•
P06702	S100A9		•	•	•	•
P29508	Serpin B3		•	•	•	•
P31947	14-3-3 protein sigma		•			•
P05141	ADP/ATP translocase 2	Ctrl/SM: M_1_-loss +N-term.-Acetyl.	•			•
P04080	Cystatin B	SM: N-term.-Acetyl.	•			•
P02788	Lactotransferrin		•			•
Q9BYD5	Cornifelin		•	•	•	
P01037	Cystatin SN		•	•	•	
P01036	Cystatin S		•		•	
P12273	Prolactin-inducible protein (PIP)		•	•		•
P63104	14-3-3 protein zeta/delta	Ctrl: N-term.-Acetyl.	•			
P59998	Actin-related protein 2/3 complex subunit 4 (ARPC4)		•			
P08311	Cathepsin G		•			
O95833	Chloride intracellular channel protein 3		•			
P23528	Cofilin-1	Ctrl: M_1_-loss +N-term.-Acetyl.	•			
O43240	Kallikrein-10		•			
P61626	Lysozyme C		•			
P35232	Prohibitin		•			
P13489	Ribonuclease inhibitor		•			
P30740	Leukocyte elastase inhibitor (Serpin B1)			•	•	•
P00558	Phosphoglycerate kinase 1			•	•	•
P02812	Basic salivary proline-rich protein 2 (bPRP-2L)			•	•	
P02814	Submaxillary gland androgen-regulated protein 3B (P-B peptide)			•	•	
P20160	Azurocidin					•
Q01469	Fatty acid-binding protein 5 (FABP-5)	SM: M_1_-loss +N-term.-Acetyl.				•
P15104	Glutamine synthetase					•
P14923	Junction plakoglobin					•
P59665	Neutrophil defensin 1					•
Q13835	Plakophilin-1					•
P05109	S100A8					•
P62987	Ubiquitin-60S ribosomal protein L40					•
P21796	Voltage-dependent anion-selective channel protein 1					•
P04083	Annexin A1	SM: M_1_-loss +N-term.-Acetyl. Phospho-S_37_		•		•
P31944	Caspase-14			•		
P20930	Filaggrin			•		
Q86YZ3	Hornerin			•		
P07237	Protein disulfide-isomerase (PDI)			•		
P68371	Tubulin beta-4B chain			•		
P25311	Zinc-alpha-2-glycoprotein			•		
P12429	Annexin A3				•	•
P52907	F-actin-capping protein subunit alpha-1	SM: M_1_-loss +N-term.-Acetyl.			•	•
P00338	L-lactate dehydrogenase A chain (LDH)	SM/SM−C: M_1_-loss +N-term- Acetyl			•	•
P29401	Transketolase				•	•
P81605	Dermcidin				•	
P04406	Glyceraldehyde-3-phosphate dehydrogenase (GAPDH)				•	
P15515	Histatin-1 (Hst-1)				•	
Q8TAX7	Mucin 7				•	
Q6UWP8	Suprabasin				•	
P07951	Tropomyosin beta chain				•	
P08670	Vimentin				•	

**Table 2:** Term ID, Term, FDR, associated genes and Uniprot-KB code of the functional network involving the protein interactors identified in the salivary cystatin D-C_26_ interactome in Ctrls using the Gene Ontology (GO) biological process and Reactome pathway databases. Only the enriched term with an FDR < 0.05 are indicated. The biological processes associated with proteins commonly detected in all the groups are highlighted in light blue color.

Term ID	Term	FDR *	Associated Genes	Uniprot KB Code
Gene Ontology (GO) Biological Process				
GO:0001895	Retina homeostasis	0.00028	*CST4, LTF, LYZ, PIP, PIGR*	P01036, P02788, P61626, P12273, P01833
GO:0060249	Anatomical structure homeostasis	0.0019	*CST4, LTF, LYZ, PIP, PIGR, ALDOA*	P01036, P02788, P61626, P12273, P01833
GO:0048871	Multicellular organismal homeostasis	0.0049	*CST4, LTF, LYZ, PIP, SFN, PIGR*	P01036, P02788, P61626, P12273, P31947, P01833
GO:0065008	Regulation of biological quality	0.0043	*CTSG, CST4, LTF, LYZ, PIP, SFN, PIGR, S100A9, SLC25A5, DSP, YWHAZ, ARPC4, CFL1, PHB, ALDOA*	P08311, P01036, P02788, P61626, P12273, P31947, P01833, P06702, P05141, P15924, P63104, P59998, P23528, P35232, P04075
GO:2000117	Negative regulation of cysteine-type endopeptidase activity	0.00028	*CST4, LTF, SERPINB3, CSTB, CST1, CST5, SFN*	P01036, P02788, P29508, Q76LA1, P01037, P28325, P31947
GO:0052548	Regulation of endopeptidase activity	0.00028	*CST4, LTF, SERPINB3, CSTB, CST1, CST5, SFN, S100A9*	P01036, P02788, P29508, Q76LA1, P01037, P28325, P31947, P06702
GO:0051336	Regulation of hydrolase activity	0.0024	*CST4, LTF, SERPINB3, CSTB, CST1, CST5, SFN, S100A9, PHB*	P01036, P02788, P29508, P04080, P01037, P28325, P31947, P06702, P35232
GO:0001580	Detection of chemical stimulus involved in sensory perception of bitter taste	0.00039	*CST4, PIP, CST1, PIGR*	P01036, P12273, P01037, P01833
GO:0019730	Antimicrobial humoral response	0.0015	*CTSG, LTF, LYZ, S100A9, DMBT1*	P08311, P02788, P61626, P06702, Q9UGM3
GO:0050829	Defense response to Gram-negative bacterium	0.0056	*CTSG, LTF, LYZ, DMBT1*	P01036, P02788, P61626, Q9UGM3
GO:0051702	Biological process involved in interaction with symbiont	0.0081	*CTSG, LTF, CFL1, PHB*	P01036, P02788, P06702, P05141
Reactome Pathway				
HSA-168249	Innate Immune System	3.13 × 10^−6^	*CTSG, LTF, LYZ, SERPINB3, CSTB, PIGR, S100A9, DSP, ARPC4, CFL1, MUC5B, ALDOA*	P01036, P02788, P61626, P29508, P04080, P01833, P06702, P15924, P59998, P06702, Q9HC84, P15924,
HSA-6798695	Neutrophil degranulation	5.02 × 10^−6^	*CTSG, LTF, LYZ, SERPINB3, CSTB, PIGR, S100A9, DSP, ALDOA*	P01036, P02788, P61626, P29508, P04080, P01833, P06702, P15924, P15924,
HSA-6803157	Antimicrobial peptides	0.0028	*CTSG, LTF, LYZ, S100A9*	P01036, P02788, P61626, P06702,
HSA-195258	RHO GTPase Effectors	0.0142	*SFN, S100A9, YWHAZ, ARPC4, CFL1*	P31947, P06702, P63104, P59998, P06702
HSA-6799990	Metal sequestration by antimicrobial proteins	0.0164	*LTF, S100A9*	P02788, P06702

* Corrected with Bonferroni step down.

**Table 3:** Term ID, Term, FDR, associated genes and Uniprot-KB code of the functional network involving the protein interactors identified in the salivary cystatin D-C_26_ interactome in SM−C using the Gene Ontology (GO) biological process and Reactome pathway databases. Only the enriched term with an FDR < 0.05 are indicated. The biological processes associated with proteins commonly detected in all the groups are highlighted in light blue.

Term ID	Term	FDR *	Associated Genes	Uniprot KB Code
Gene Ontology (GO) Biological Process				
GO:0061844	Antimicrobial humoral immune response mediated by antimicrobial peptide	0.00014	*S100A9, DMBT1, GAPDH, DCD, MUC7, HTN1*	P06702, Q9UGM3, P04406, P81605, Q8TAX7, P15515
GO:0031640	Killing of cells of another organism	0.0022	*GAPDH, DCD, MUC7, HTN1*	P04406, P81605, Q8TAX7, P15515
GO:0050832	Defense response to fungus	0.0020	*S100A9, GAPDH, DCD, HTN1*	P06702, P04406, P81605, P04075
GO:0098542	Defense response to other organism	0.0150	*ANXA3, S100A9, DMBT1, GAPDH, DCD, MUC7, HTN1, VIM*	P12429, P06702, Q9UGM3, P04406, P81605, Q8TAX7, P15515, P08670
GO:0042742	Defense response to bacterium	0.0215	*ANXA3, S100A9, DMBT1, DCD, HTN1*	P12429, P06702, Q9UGM3, Q9UGM3, P81605, P04075
GO:0044419	Biological process involved in interspecies interaction between organisms	0.0316	*ANXA3, SERPINB3, S100A9, DMBT1, GAPDH, DCD, MUC7, HTN1, VIM*	P12429, P29508, P06702, Q9UGM3, P04406, P81605, Q8TAX7, P15515, P08670
GO:0052548	Regulation of endopeptidase activity	0.00019	*CST4, SERPINB3, CST1, CST5, S100A9, SERPINB1, GAPDH, SMR3B*	P01036, P29508, P01037, P28325, P06702, P30740, P04406, P02814
GO:0006096	Glycolytic process	0.00091	*PGK1, GAPDH, LDHA, ALDOA*	P00558, P04406, P00338, P04075
GO:0035606	Peptidyl-cysteine S-trans-nitrosylation	0.0063	*S100A9, GAPDH*	P06702, P04406
GO:0001580	Detection of chemical stimulus involved in sensory perception of bitter taste	0.0113	*CST4, CST1, PIGR*	P01036, P01037, P01833
GO:0006955	Immune response	0.0150	*ANXA3, PIGR, S100A9, DMBT1, GAPDH, DCD, MUC7, HTN1, VIM*	P12429, P01833, P06702, Q9UGM3, P04406, P81605, Q8TAX7, P15515, P08670
GO:0035425	Autocrine signaling	0.0253	*SERPINB3, S100A9*	P29508, P06702
Reactome Pathway				
HSA-168249	Innate Immune System	7.93 × 10^−5^	*CAPZA1, SERPINB3, PIGR, S100A9, DSP, SERPINB1, DCD, MUC7, HTN1, MUC5B, ALDOA*	P52907, P29508, P01833, P01833, P15924, P30740, P81605, Q8TAX7, P15515, Q9HC84, P04075,
HSA-70263	Gluconeogenesis	0.0107	*PGK1, GAPDH, ALDOA*	P00558, P04406, P04075
HSA-6798695	Neutrophil degranulation	0.0179	*SERPINB3, PIGR, S100A9, DSP, SERPINB1, ALDOA*	P01833, P01833, P06702, P15924, P30740, P04075

* Corrected with Bonferroni step down.

**Table 4:** Term ID, Term, FDR, associated genes and Uniprot-KB code of the functional network involving the protein interactors identified in the salivary cystatin D-C_26_ interactome in SM+C using the Gene Ontology (GO) biological process and Reactome pathway databases. Only the enriched term with an FDR < 0.05 are indicated. The biological processes associated with proteins commonly detected in all the groups are highlighted in light blue.

Term ID	Term	FDR *	Associated Genes	Uniprot KB code
Gene Ontology (GO) Biological Process				
GO:0030216	Keratinocyte differentiation	0.00015	*CNFN, FLG, HRNR, ANXA1, DSP, CASP14*	Q9BYD5, P20930, Q86YZ3, P04083, P15924, P31944
GO:0030855	Epithelial cell differentiation	0.00072	*CNFN, FLG, HRNR, DMBT1, PGK1, ANXA1, DSP, CASP14*	Q9BYD5, P20930, Q86YZ3, Q9UGM3, P00558, P04083, P15924, P31944
GO:0018149	Peptide cross-linking	0.0121	*FLG, ANXA1, DSP*	P20930, P04083, P15924
GO:0001580	Detection of chemical stimulus involved in sensory perception of bitter taste	0.00063	*PIP, AZGP1, CST1, PIGR*	P12273, P25311, P01037, P01833
GO:0051346	Negative regulation of hydrolase activity	0.0032	*SERPINB3, CST1, CST5, ANXA1, SERPINB1, SMR3B*	P29508, P01037, P28325, P04083, P30740, P02814
GO:0052548	Regulation of endopeptidase activity	0.0072	*SERPINB3, CST1, CST5, S100A9, SERPINB1, SMR3B*	P30740, P01037, P28325, P06702, P02814
GO:0035425	Autocrine signaling	0.0371	*SERPINB3, S100A9*	P29508, P06702
GO:0048871	Multicellular organismal homeostasis	0.0413	*PIP, AZGP1, PIGR, FLG, HRNR*	P12273, P25311, P01833, P11362, Q86YZ3
Reactome Pathway				
HSA-6798695	Neutrophil degranulation	6.70 × 10^−5^	*SERPINB3, TUBB4B, PIGR, S100A9, HRNR, DSP, SERPINB1, ALDOA*	P29508, P68371, P01833, P06702, Q86YZ3, P15924, P30740, V9HWH1, P04075
HSA-168256	Immune System	0.0020	*SERPINB3, P4HB, TUBB4B, PIGR, S100A9, HRNR, ANXA1, DSP, SERPINB1, MUC5B, ALDOA*	P29508, P07237, P68371, P01833, P06702, Q86YZ3, P04083, P15924, P30740, Q9HC84, P04075

* Corrected with Bonferroni step down.

**Table 5:** Age/gender, diagnosis, presence of oral or gastrointestinal symptoms, tryptase serum level and eventual mutation of c-KIT receptor of each patient at the time of the study.

Patient	Age/Gender	Diagnosis	Secondary Cutaneous Symptoms (SM+C)	Tryptase (μg/L)	Mutation
#9	45/M	SM	Y	33.6	M541L
#11	58/F	SM	Y	20.2	D816V
#13	35/F	SM	Y	44.7	
#15	38/F	SM	Y	7.66	D816V
#16	52/M	SM	Y	37.5	D816V
#18	55/M	SM		27.1	D816V
#19	48/M	SM	Y	16.2	
#20	32/M	SM	Y	53.5	D816V
#22	60/F	SM	Y	23.6	
#24	49/M	SM	Y	29.1	D816V
#26	35/F	SM	Y	16.5	
#28	38/F	SM	Y	26.2	D816V
#29	57/M	SM	Y	77.4	D816V
#35	34/M	SM	Y	28.7	
#36	52/F	SM	Y	16.1	D816V
#41	38/M	SM	Y	26.3	
#49	51/M	SM	Y	5.02	
#51	29/M	SM	Y	64.5	
#59	67/F	SM	Y	30.6	D816V
#60	49/F	SM	Y	15.8	D816V
#63	56/M	SM	Y	60.8	D816V
#66	36/F	SM	Y	6.63	
#68	42/M	SM		21.9	D816V
#68	54/F	SM	Y	21.9	D816V
#70	72/M	SM	Y	59.7	D816V
#71	35/M	SM		16.5	
#72	44/F	SM	Y	126	D816V
#73	35/M	SM	Y	40.8	D816V
#74	56/M	SM		5.67	
#75	64/F	SM	Y	45.1	
#76	79/F	SM	Y	73.9	D816V
#77	35/F	SM	Y	44.7	

## Data Availability

HR-MS/MS data are freely available at the ProteomeXchange Consortium (http://ww.ebi.ac.uk/pride (accessed on 11 January 2023)) with the dataset identifier PXD042567.

## Supplementary Materials

The following supporting information can be downloaded at: https://www.mdpi.com/article/10.3390/ijms241914613/s1.
