# Supplementary material for: Salivary Cystatin D Interactome in Patients with Systemic Mastocytosis: An Exploratory Study
Source: Int J Mol Sci. 2023 Sep 27;24(19):14613. doi: 10.3390/ijms241914613 (PMC10572539; doi:10.3390/ijms241914613)
Supplement: Supplementary file 1 [file ijms-24-14613-s001.zip › ijms-2453636-supplementary.pdf]

Figure S1. Western blot and immunodetection with cystatin D-C_{26} Ab of whole saliva from healthy control subjects separated in SDS-PAGE under non reducing (NR) and reducing (R) conditions. In panel (a) is possible to highlight a signal at MW > 250, that disappear in R condition (b).

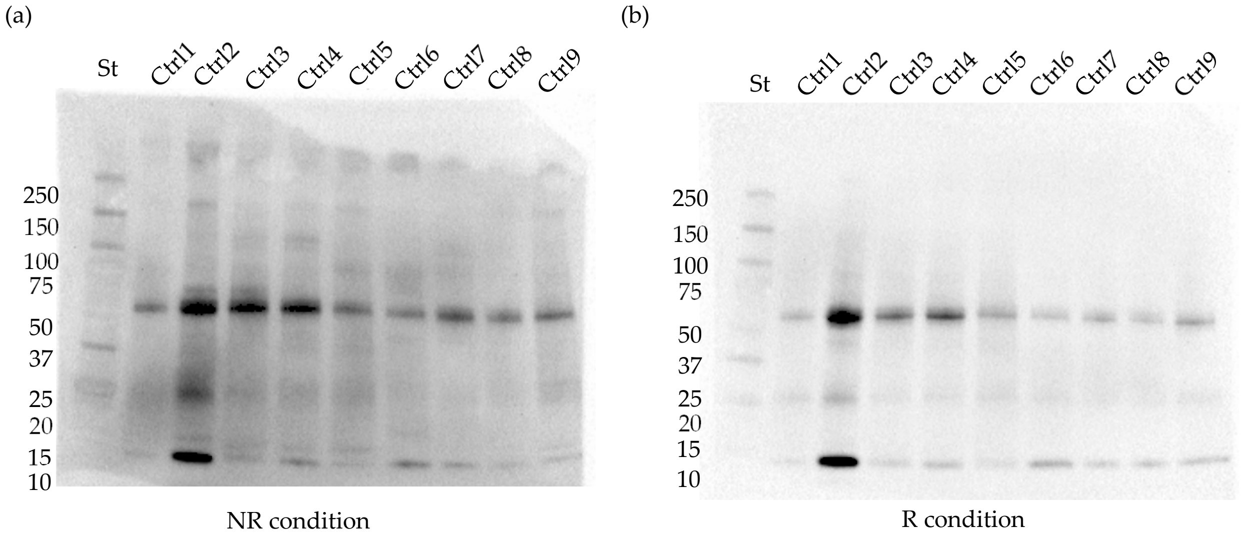

Figure S2. SDS-PAGE gels under R conditions used to prepare slices for the mass spectrometry (MS) bottom-up analysis. Co-IPs obtained from salivary pools of Ctrls, SM-C, SM+C1, SM+C2, SM1, SM2 and NEG were loaded in the wells of the two gels in duplicate as indicated (6 µg of total protein amount for each sample). Ctrl samples were loaded in duplicate in each of the two gels, but only the lane indicated with an asterisk were used for the MS bottom-up analysis. Samples St: molecular weight standards.

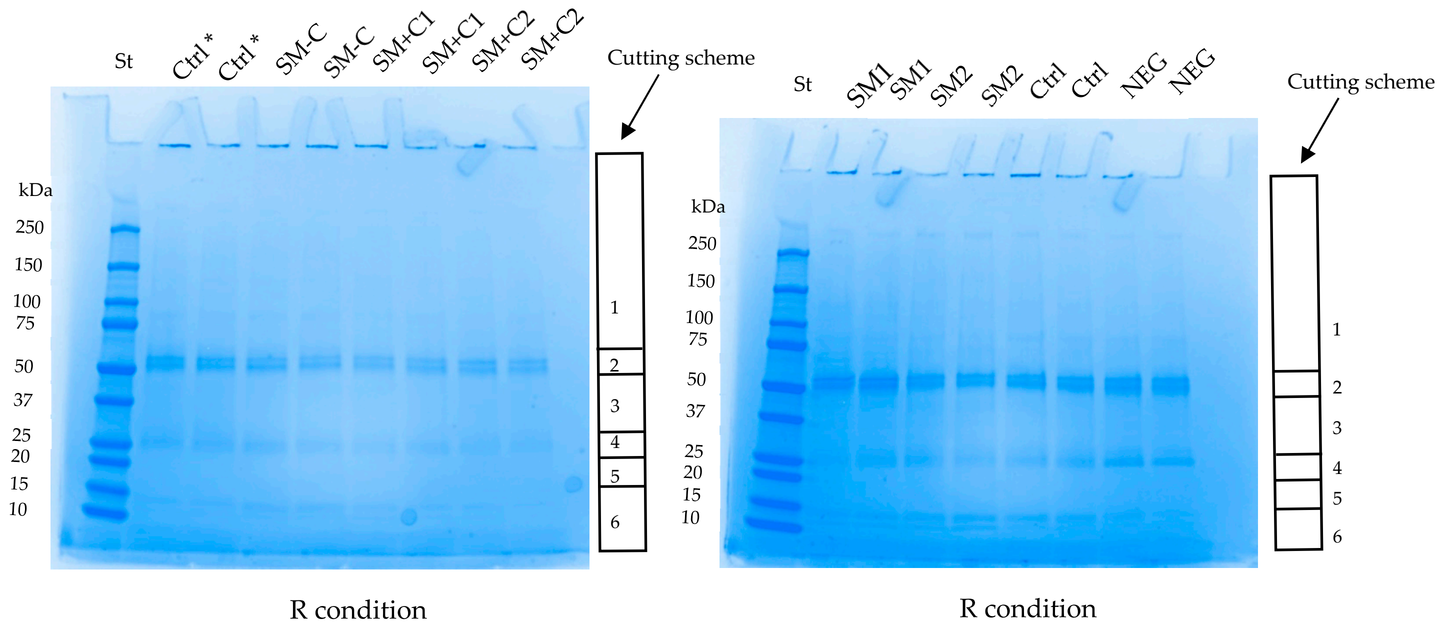

Figure S3. Chart of comparison of the total protein LFQ abundances (Log10) determined by PD software in patients and controls. Non normalized LFQ abundances are reported in panel (a), LFQ abundances normalized on the total peptide amount measured in the samples are reported in panel (b).

(a)

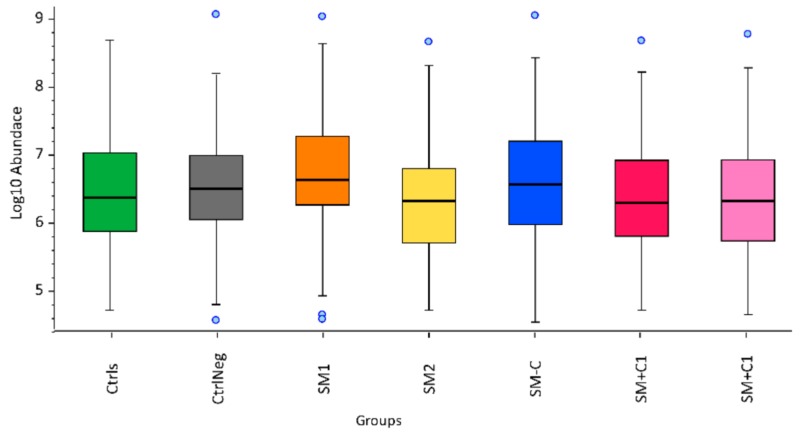

(b)

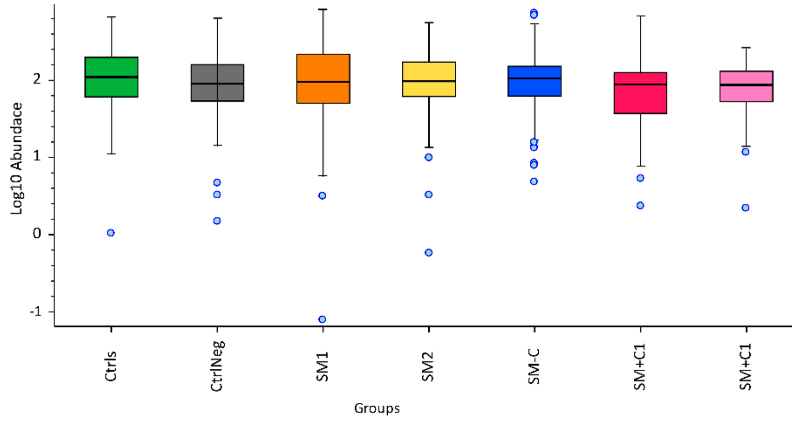

Table S1: Results of the comparison of the protein LFQ abundance among Ctrls, SM, SM+C, SM-C and NEG groups by student t-test. LFQ abundances and fold change (FC) are indicated, when a FC ≤ -2 was obtained with respect to NEG the protein/peptide was considered included among the cystatin D-C_{26} interactors. In italic are indicated the excluded proteins based on the NEG comparison.

UNiprot-KB codes	Description	LFQ Abundances					FC	FC	FC	FC
		NEG	Ctrl	SM	SM+C	SM-C	vs Ctrl	vs SM	vs SM+C	vs SM-C
P31947	14-3-3 protein sigma	1.58E+07	6.21E+07	3.75E+07	1.70E+07	2.09E+07	-3.9	-2.3	0.5	-0.8
P63104	14-3-3 protein zeta/delta	1.75E+07	4.08E+07	2.22E+07	1.43E+07	1.35E+07	-2.4	-0.1	0.9	0.7
P59998	Actin-related protein 2/3 complex subunit 4	5.99E+05	2.95E+06			6.98E+04	-4.6	18.5	18.5	6.2
P05141	ADP/ATP translocase 2	1.28E+06	3.05E+06	3.35E+06		2.48E+05	-2.5	-2.3	20.6	4.7
P02768	*Albumin*	*3.28E+08*	*2.89E+08*	*1.78E+08*	*3.34E+08*	*3.98E+08*	*0.4*	*1.8*	*0.0*	*-0.6*
P0DUB6	*Alpha-amylase 1A*	*1.01E+08*	*5.75E+07*	*6.60E+07*	*1.24E+08*	*1.02E+08*	*1.6*	*1.2*	*-0.5*	*0.0*
P04083	Annexin A1	1.62E+08	1.95E+08	4.94E+08	4.32E+08		-0.5	-3.2	-2.7	0.0
P07355	*Annexin A2*	*1.69E+08*	*1.64E+08*	*1.37E+08*	*1.96E+08*	*2.12E+08*	*0.1*	*0.6*	*-0.4*	*-0.7*
P12429	Annexin A3	7.90E+04		4.86E+06		6.06E+06	0.0	-11.2	12.6	-12.5
P02647	*Apolipoprotein A-I*	*9.21E+06*	*4.31E+06*				*2.2*	*26.3*	*26.3*	*26.3*
P05089	*Arginase-1*	*2.50E+07*	*1.76E+07*	*1.45E+07*	*2.46E+07*	*1.69E+07*	*1.0*	*2.7*	*0.0*	*1.1*
P06576	*ATP synthase subunit beta, mitochondrial*	*1.29E+06*	*2.00E+05*			*2.40E+06*	*5.4*	*20.7*	*20.7*	*-1.8*
P20160	Azurocidin	6.16E+06		1.29E+07	4.35E+06	9.84E+06	4.5	-2.1	1.1	-1.4
P02812	Basic salivary proline-rich protein 2				1.11E+07	3.67E+08	0.0	0.0	-26.9	-37.0
Q96DR5	*BPI fold-containing family A member 2*	*3.01E+07*	*3.34E+07*	*4.53E+06*		*2.11E+07*	*-0.3*	*7.1*	*29.8*	*1.0*
Q8TDL5	*BPI fold-containing family B member 1*	*1.40E+07*	*3.55E+06*	*6.34E+06*		*1.10E+07*	*4.0*	*2.6*	*27.5*	*0.7*
Q8N4F0	*BPI fold-containing family B member 2*	*1.74E+07*	*1.23E+07*		*6.68E+06*	*3.31E+07*	*1.0*	*28.2*	*2.8*	*-1.9*
P23280	*Carbonic anhydrase 6*	*1.32E+08*	*6.83E+07*	*6.53E+07*	*4.36E+07*	*1.17E+08*	*1.9*	*3.1*	*3.2*	*0.3*
P31944	Caspase-14	5.33E+06	2.54E+06		1.37E+07	5.41E+06	2.1	24.8	-2.7	0.0
P08311	Cathepsin G	6.58E+07	1.50E+08	9.64E+07		7.27E+07	-2.4	-0.8	32.0	-0.3
O95833	Chloride intracellular channel protein 3	4.55E+05	1.19E+06				-2.8	17.7	17.7	17.7
P23528	Cofilin-1	1.78E+06	5.56E+06	1.31E+06	1.90E+06	1.09E+06	-3.3	1.9	0.0	1.4
Q9BYD5	Cornifelin		1.14E+07		2.10E+06	1.79E+06	-27.0	0.0	-21.9	-21.6
P04080	Cystatin-B	7.89E+05	2.61E+06	3.22E+06	1.61E+06	7.62E+05	-3.5	-4.1	-1.9	0.1

UNiprot-KB codes	Description	LFQ Abundances					FC	FC	FC	FC
		NEG	Ctrl	SM	SM+C	SM-C	vs Ctrl	vs SM	vs SM+C	vs SM-C
P28325	Cystatin-D	3.56E+06	4.67E+07	2.00E+07	9.69E+06	2.06E+07	-7.4	-4.9	-2.8	-5.1
P01036	Cystatin-S		7.11E+06			1.24E+06	-25.6	0.0	0.0	-20.6
P01037	Cystatin-SN	2.27E+06	4.43E+07	3.78E+06	6.86E+06	8.33E+06	-8.6	-1.4	-2.7	-3.8
P54108	*Cysteine-rich secretory protein 3*	*2.81E+06*	*2.38E+05*		*2.64E+06*	*8.79E+05*	*7.1*	*22.9*	*0.3*	*3.4*
Q9UGM3	Deleted in malignant brain tumors 1 protein -	1.36E+07	7.13E+07	2.26E+08	3.49E+07	3.43E+07	-4.8	-7.3	-2.4	-2.7
P81605	Dermcidin	4.82E+07	5.05E+07	3.14E+07	7.18E+07	1.25E+08	-0.1	2.0	-1.1	-2.7
Q08554	*Desmocollin-1*	*6.11E+06*	*2.55E+06*		*8.39E+06*	*1.04E+07*	*2.5*	*25.2*	*-0.9*	*-1.5*
Q02413	*Desmoglein-1*	*2.27E+07*	*3.09E+07*	*1.92E+07*	*3.21E+07*	*3.83E+07*	*-0.9*	*0.6*	*-1.0*	*-1.5*
P15924	Desmoplakin	2.00E+07	6.91E+07	4.35E+07	4.32E+07	3.68E+07	-3.6	-2.2	-2.2	-1.8
P52907	F-actin-capping protein subunit alpha-1	1.15E+06	1.69E+06	5.32E+06	1.88E+06	3.00E+06	-1.1	-4.1	-1.2	-2.8
Q01469	Fatty acid-binding protein 5	1.96E+07	2.97E+07	4.24E+07	1.06E+07	2.09E+07	-1.2	-2.1	2.2	-0.2
P20930	Filaggrin	4.65E+06	7.66E+06		1.15E+07	8.09E+06	-1.4	24.4	-2.5	-1.6
Q5D862	Filaggrin-2	2.29E+07	1.80E+07	1.29E+07		6.20E+06	0.7	1.6	29.0	3.8
P04075	Fructose-bisphosphate aldolase A	4.89E+06	1.33E+07	2.15E+07	3.82E+07	4.48E+07	-2.9	-4.2	-5.3	-6.4
P47929	*Galectin-7*	*5.25E+06*	*6.91E+06*		*9.01E+06*	*3.21E+06*	*-0.8*	*24.7*	*-1.4*	*1.4*
P15104	Glutamine synthetase	1.33E+05	2.76E+05	2.17E+06			3.4	-8.0	0.0	14.1
P09211	*Glutathione S-transferase P*	*1.80E+07*	*1.90E+07*	*1.16E+07*	*7.50E+06*	*4.79E+06*	*-0.2*	*1.4*	*3.0*	*3.8*
P04406	Glyceraldehyde-3-phosphate dehydrogenase	1.64E+07	1.25E+07	2.35E+07	2.67E+07	3.45E+07	0.8	-0.9	-1.4	-2.1
P04792	*Heat shock protein beta-1*	*6.37E+07*	*7.30E+07*	*6.94E+07*	*4.09E+07*	*3.17E+07*	*-0.4*	*-0.2*	*1.3*	*2.0*
P15515	Histatin-1					2.83E+06	0.0	0.0	0.0	-22.9
Q86YZ3	Hornerin	1.99E+08	3.24E+08	1.04E+08	4.49E+08	2.93E+08	-1.4	2.2	-2.3	-1.1
P14923	Junction plakoglobin	1.66E+07	2.76E+07	4.67E+07	2.42E+07	1.52E+07	-1.5	-2.8	-1.1	0.3
O43240	Kallikrein-10	2.39E+06	6.21E+06	1.59E+06		1.95E+06	-2.8	1.2	22.4	0.6
P00338	L-lactate dehydrogenase A chain	1.17E+06	5.97E+05	3.66E+06		3.92E+06	1.9	-3.0	20.4	-3.5
P02788	Lactotransferrin	3.05E+05	4.70E+06	1.69E+07		2.17E+06	-7.9	-11.0	16.5	-5.7
P30740	Leukocyte elastase inhibitor	5.22E+06	1.76E+06	2.13E+07	1.81E+07	2.09E+07	3.1	-4.0	-3.4	-4.0
P61626	Lysozyme C	3.15E+07	1.17E+08	5.30E+07	5.31E+07	5.72E+07	-3.8	-1.3	-1.5	-1.7

UNiprot-KB codes	Description	LFQ Abundances					FC	FC	FC	FC
		NEG	Ctrl	SM	SM+C	SM-C	vs Ctrl	vs SM	vs SM+C	vs SM-C
Q9HC84	Mucin-5B		1.59E+08	1.76E+07	4.45E+06	3.93E+06	-34.6	-27.1	-22.4	-23.9
Q8TAX7	Mucin-7	8.51E+06	1.35E+07	5.93E+06		1.81E+07	-1.3	1.6	26.1	-2.2
P24158	*Myeloblastin*	*1.72E+07*	*2.54E+07*	*6.19E+06*		*4.05E+06*	*-1.1*	*3.0*	*28.1*	*4.2*
P05164	*Myeloperoxidase*	*1.77E+07*	*1.37E+07*	*8.55E+06*	*3.14E+07*	*2.28E+07*	*0.7*	*2.4*	*-1.4*	*-0.7*
P59665	Neutrophil defensin 1	1.44E+08	2.91E+07	5.25E+08	9.21E+07	1.46E+08	4.6	-3.0	1.3	0.0
P08246	*Neutrophil elastase*	*2.12E+07*	*3.28E+07*	*1.61E+07*		*9.55E+06*	*-1.3*	*0.8*	*28.7*	*2.3*
P62937	*Peptidyl-prolyl cis-trans isomerase A*	*8.42E+06*	*8.50E+06*	*1.02E+07*		*8.23E+06*	*0.0*	*0.1*	*26.1*	*0.1*
Q06830	*Peroxiredoxin-1*	*3.59E+06*	*5.66E+06*	*8.61E+05*		*3.01E+06*	*-1.3*	*4.4*	*23.6*	*0.5*
P30041	*Peroxiredoxin-6*	*2.10E+06*	*2.76E+06*				*-0.8*	*22.1*	*22.1*	*22.1*
P00558	Phosphoglycerate kinase 1	1.27E+06	2.18E+06	7.49E+06	3.91E+06	1.18E+07	-1.6	-5.0	-3.2	-6.4
P18669	*Phosphoglycerate mutase 1*	*4.17E+06*	*5.57E+06*	*4.46E+06*		*2.37E+06*	*-0.8*	*-0.2*	*24.1*	*1.6*
Q13835	Plakophilin-1	2.41E+06	3.62E+06	1.09E+07		4.14E+05	-1.2	-4.3	22.5	5.1
P01833	Polymeric immunoglobulin receptor	2.10E+07	1.26E+08	1.25E+08	7.03E+07	1.03E+08	-5.2	-4.9	-3.2	-4.6
P35232	Prohibitin	5.70E+05	1.34E+06	0.00E+00		9.53E+05	-2.5	18.3	18.3	-1.5
P12273	Prolactin-inducible protein	4.75E+07	3.86E+08	7.10E+07	1.03E+08	7.81E+07	-6.0	-0.9	-2.1	-1.4
P30101	*Protein disulfide-isomerase A3*	*2.52E+06*		*1.71E+06*	*1.15E+06*		*22.6*	*1.1*	*2.7*	*22.6*
P07237	Protein disulfide-isomerase				1.14E+06		0.0	0.0	-20.1	0.0
P05109	Protein S100-A8	6.52E+07	3.26E+07	1.96E+08	4.06E+07	4.38E+07	2.0	-3.0	1.5	1.1
P06702	Protein S100-A9	7.05E+06	1.04E+08	2.78E+07	4.57E+07	4.48E+07	-7.8	-3.8	-5.3	-5.3
Q08188	*Protein-glutamine gamma-glutamyltransferase E*	*7.79E+06*				*8.32E+06*	*0.0*	*25.9*	*25.9*	*-0.2*
P14618	*Pyruvate kinase PKM*	*6.63E+06*	*6.95E+06*		*4.07E+06*	*2.68E+06*	*-0.1*	*25.4*	*1.6*	*2.6*
P13489	Ribonuclease inhibitor	4.57E+05	1.49E+06				-3.4	17.7	17.7	3.8
P29508	Serpin B3	3.07E+06	7.88E+06	7.37E+06	1.25E+07	9.49E+06	-2.7	-2.5	-3.9	-3.3
P02814	Submaxillary gland androgen-regulated protein 3B	5.30E+05	3.73E+05		4.71E+06	2.64E+08	1.0	18.1	-6.3	-17.9
Q6UWP8	Suprabasin	2.67E+06	1.93E+06			2.69E+07	0.9	22.8	22.8	-6.7
P29401	Transketolase			4.22E+06		2.72E+06	0.0	-23.6	0.0	-22.8
P60174	*Triosephosphate isomerase*	*9.50E+06*	*4.26E+06*	*2.61E+06*	*1.14E+07*	*3.53E+06*	*2.3*	*6.4*	*-0.3*	*2.9*

UNiprot-KB codes	Description	LFQ Abundances					FC	FC	FC	FC
		NEG	Ctrl	SM	SM+C	SM-C	vs Ctrl	vs SM	vs SM+C	vs SM-C
P07951	Tropomyosin beta chain	3.16E+05				2.68E+06	16.6	16.6	3.7	-6.2
Q71U36	Tubulin alpha-1A chain	3.24E+06	1.13E+06		2.92E+06	3.82E+06	3.0	23.3	0.3	-0.5
P68371	Tubulin beta-4B chain	2.88E+05			7.85E+05		16.3	16.3	-2.6	3.6
P62987	Ubiquitin-60S ribosomal protein L40			2.65E+06			0.0	-22.7	0.0	0.0
P08670	Vimentin					1.17E+06	0.0	0.0	0.0	-20.4
P21796	Voltage-dependent anion-selective channel protein 1			1.14E+06			0.0	-20.2	0.0	0.0
P25311	Zinc-alpha-2-glycoprotein	5.82E+06	3.18E+06		1.69E+07	7.40E+06	1.7	25.0	-3.0	-0.7
Q96DA0	*Zymogen granule protein 16 homolog B*	*1.86E+08*	*2.67E+08*	*7.14E+07*	*5.46E+07*	*1.55E+07*	*-1.0*	*2.9*	*3.8*	*7.2*

Table S2. Main MS data concerning the identification of proteins/peptides of the multi-protein complex (mPC) immunoprecipitated with cystatin D-C_{26} from salivary pool of Ctrl samples.

**Uniprot-KB code**	**Description (PTM)**	**Sum PEP Score**	**Coverage (%)**	**# Peptides**	**# Unique Peptides**	**# AAs**	**MW (kDa)**	**Score Sequest HT**	**Gene Symbol**
P31947	14-3-3 protein sigma	51.6	72	11	11	248	27.8	49.0	SFN
P63104	14-3-3 protein zeta/delta **(N-term.-Acetyl.)**	55.6	59	10	9	245	27.7	107.7	YWHAZ
P59998	Actin-related protein 2/3 complex subunit 4	2.3	5	1	1	168	19.7	0.0	ARPC4
P05141	ADP/ATP translocase 2 **(M_1-loss +N-term.-Acetyl.)**	12.5	8	2	2	298	32.8	10.9	SLC25A5
P08311	Cathepsin G	48.605	47	12	12	255	28.8	38.53	CTSG
O95833	Chloride intracellular channel protein 3	4.8	12	2	2	236	26.6	6.7	CLIC3
P23528	Cofilin-1 **(M_1-loss +N-term.-Acetyl.)**	11.1	33	3	3	166	18.5	31.8	CFL1
Q9BYD5	Cornifelin	9.3	44	2	2	112	12.4	25.5	CNFN
P04080	Cystatin-B	5.6	34	2	2	98	11.1	2.5	CSTB
P28325	Cystatin-D	19.9	39	3	3	142	16.1	116.8	CST5
P01036	Cystatin-S	15.0	60	5	4	141	16.2	24.1	CST4
P01037	Cystatin-SN	28.4	49	6	5	141	16.4	47.5	CST1
Q9UGM3	Deleted in malignant brain tumors 1 protein	42.0	25	7	7	2413	260.6	62.7	DMBT1
P15924	Desmoplakin	51.5	7	15	15	2871	331.6	59.9	DSP
P04075	Fructose-bisphosphate aldolase A **(M_1-loss)**	19.896	18	6	6	364	39.4	19.94	ALDOA
O43240	Kallikrein-10	7.1	10	2	2	276	30.2	2.6	KLK10
P02788	Lactotransferrin	6.8	5	3	3	710	78.1	4.3	LTF
P61626	Lysozyme C	23.9	54	7	7	148	16.5	80.1	LYZ
Q9HC84	Mucin-5B	13.4	2	3	3	5762	596	14.9	MUC5B
P01833	Polymeric immunoglobulin receptor	61.5	22	13	13	764	83.2	92.2	PIGR
P35232	Prohibitin	1.8	4	1	1	272	29.8	0.0	PHB
P12273	Prolactin-inducible protein	49.254	71	10	10	146	16.6	67.26	PIP
P06702	Protein S100-A9	21.9	54	5	5	114	13.2	29.5	S100A9
P13489	Ribonuclease inhibitor	2.4	2	1	1	461	49.9	5.9	RNH1
P29508	Serpin B3	9.2	7	2	2	390	44.5	12.8	SERPINB3

Table S3. Main MS data concerning the identification of proteins/peptides of the multi-protein complex (mPC) immunoprecipitated with cystatin D-C_{26} from salivary pool of NEG samples.

Uniprot-KB code	Description (PTM)	Sum PEP Score	Coverage (%)	# Peptides	# Unique Peptides	# AAs	MW (kDa)	Score Sequest HT	Gene Symbol
P31947	14-3-3 protein sigma	6.531	14	2	2	248	27.8	6.36	SFN
P63104	14-3-3 protein zeta/delta **(N-term-Acetyl.)**	27.958	47	7	7	245	27.7	42.66	YWHAZ
P52209	6-phosphogluconate dehydrogenase, decarboxylating	6.152	6	2	2	483	53.1	6.27	PGD
P05141	ADP/ATP translocase 2 **(M_1-loss +N-term- Acetyl)**	5.283	8	1	1	298	32.8	6.82	SLC25A5
P02768	Albumin	32.549	18	11	11	609	69.3	83.07	ALB
P0DUB6	Alpha-amylase 1A	43.378	32	12	12	511	57.7	75.29	AMY1A
P04083	Annexin A1	100.74	64	24	24	346	38.7	182.35	ANXA1
P07355	Annexin A2 **(M_1-loss +N-term- Acetyl)**	45.707	52	15	15	339	38.6	77.01	ANXA2
P02647	Apolipoprotein A-I	7.04	9	2	2	267	30.8	5.53	APOA1
P05089	Arginase-1	3.649	7	2	2	322	34.7	14.82	ARG1
P06576	ATP synthase subunit beta, mitochondrial	2.49	4	1	1	529	56.5	2.86	ATP5F1B
P20160	Azurocidin	8.825	14	3	3	251	26.9	8.43	AZU1
Q96DR5	BPI fold-containing family A member 2	6.656	18	3	3	249	27	5.14	BPIFA2
Q8TDL5	BPI fold-containing family B member 1	10.274	8	3	3	484	52.4	9.26	BPIFB1
Q8N4F0	BPI fold-containing family B member 2	37.763	22	7	7	458	49.1	42.06	BPIFB2
P23280	Carbonic anhydrase 6	23.898	25	5	5	308	35.3	88.15	CA6
P31944	Caspase-14	2.615	8	2	2	242	27.7	4.09	CASP14
P08311	Cathepsin G	34.941	38	10	10	255	28.8	27.7	CTSG
P23528	Cofilin-1 **(M_1-loss +N-term- Acetyl)**	4.553	14	2	2	166	18.5	12.23	CFL1
Q15517	Corneodesmosin	2.182	4	1	1	529	51.5	2.97	CDSN
Q9BYD5	Cornifelin	5.769	20	1	1	112	12.4	3.92	CNFN
P01037	Cystatin-SN	14.556	31	3	3	141	16.4	18.04	CST1
P54108	Cysteine-rich secretory protein 3	3.002	7	2	2	245	27.6	5.67	CRISP3
Q9UGM3	Deleted in malignant brain tumors 1 protein	14.198	20	4	4	2413	260.6	22.64	DMBT1
P81605	Dermcidin	14.788	37	5	5	110	11.3	38.92	DCD
Q08554	Desmocollin-1	5.987	3	3	3	894	99.9	6.67	DSC1

Uniprot-KB code	Description (PTM)	Sum PEP Score	Coverage (%)	# Peptides	# Unique Peptides	# AAs	MW (kDa)	Score Sequest HT	Gene Symbol
Q02413	Desmoglein-1	19.881	6	5	5	1049	113.7	57.72	DSG1
P15924	Desmoplakin	20.836	4	9	9	2871	331.6	36.16	DSP
P52907	F-actin-capping protein subunit alpha-1 3 **(M_1-loss +N-term- Acetyl)**	2.963	5	1	1	286	32.9	3.2	CAPZA1
Q01469	Fatty acid-binding protein 5	4.748	18	2	2	135	15.2	44.75	FABP5
P20930	Filaggrin	3.226	2	2	2	4061	434.9	5.21	FLG
Q5D862	Filaggrin-2	19.498	5	5	5	2391	247.9	57.64	FLG2
P04075	Fructose-bisphosphate aldolase A **(M_1-loss)**	20.254	25	7	7	364	39.4	24.18	ALDOA
P47929	Galectin-7	12.677	30	3	3	136	15.1	18.55	LGALS7; LGALS7B
P15104	Glutamine synthetase	2.784	4	1	1	373	42	3.79	GLUL
P09211	Glutathione S-transferase P **(M_1-loss)**	11.785	23	3	3	210	23.3	23.13	GSTP1
P04406	Glyceraldehyde-3-phosphate dehydrogenase	7.33	13	3	3	335	36	21.27	GAPDH
P04792	Heat shock protein beta-1	17.543	52	6	6	205	22.8	52.01	HSPB1
P14923	Junction plakoglobin	14.783	9	5	5	745	81.7	24.22	JUP
P00338	L-lactate dehydrogenase A chain	5.309	8	2	2	332	36.7	5.76	LDHA
P30740	Leukocyte elastase inhibitor	7.285	10	3	3	379	42.7	9.2	SERPINB1
P61626	Lysozyme C	13.553	28	4	4	148	16.5	56.78	LYZ
Q9HC84	Mucin-5B	3.753	1	1	1	5762	596	3.86	MUC5B
Q8TAX7	Mucin-7	3.315	3	1	1	377	39.1	2.98	MUC7
P24158	Myeloblastin	4.321	8	2	2	256	27.8	4.89	PRTN3
P05164	Myeloperoxidase	5.777	4	3	3	745	83.8	6.17	MPO
P59665	Neutrophil defensin 1	8.037	20	3	3	94	10.2	16.49	DEFA1; DEFA1B
P08246	Neutrophil elastase	10.904	18	4	4	267	28.5	14.07	ELANE
P62937	Peptidyl-prolyl cis-trans isomerase A **(Met_1-loss)**	24.279	25	4	4	165	18	16.79	PPIA
Q06830	Peroxiredoxin-1	5.483	21	3	3	199	22.1	7.5	PRDX1
P30041	Peroxiredoxin-6 **(Met_1-loss)**	4.574	17	2	2	224	25	3.78	PRDX6
P00558	Phosphoglycerate kinase 1	3.585	8	2	2	417	44.6	2.44	PGK1
P18669	Phosphoglycerate mutase 1	7.11	20	3	3	254	28.8	7.74	PGAM1
Q13835	Plakophilin-1	6.087	3	2	2	747	82.8	10.93	PKP1
P01833	Polymeric immunoglobulin receptor	18.793	8	4	4	764	83.2	57.08	PIGR

Uniprot-KB code	Description (PTM)	Sum PEP Score	Coverage (%)	# Peptides	# Unique Peptides	# AAs	MW (kDa)	Score Sequest HT	Gene Symbol
P12273	Prolactin-inducible protein	11.861	22	4	4	146	16.6	22.99	PIP
P30101	Protein disulfide-isomerase A3	4.839	5	2	2	505	56.7	5.17	PDIA3
P05109	Protein S100-A8	3.997	12	1	1	93	10.8	29.07	S100A8
P06702	Protein S100-A9	5.355	28	2	2	114	13.2	20.95	S100A9
Q04941	Proteolipid protein 2	3.221	9	1	1	152	16.7	2.82	PLP2
P14618	Pyruvate kinase PKM	5.474	5	2	2	531	57.9	13.31	PKM
P29508	Serpin B3	5.046	6	2	2	390	44.5	7	SERPINB3
Q6UWP8	Suprabasin	3.987	9	1	1	590	60.5	3.8	SBSN
P60174	Triosephosphate isomerase	15.005	40	6	6	249	26.7	23.68	TPI1
Q71U36	Tubulin alpha-1A chain	8.473	11	3	3	451	50.1	8.64	TUBA1A
Q96DA0	Zymogen granule protein 16 homolog B	27.468	45	6	6	208	22.7	106.48	ZG16B

Table S4. Main MS data concerning the identification of proteins/peptides of the multi-protein complex (mPC) immunoprecipitated with cystatin D-C26 from salivary pool of SM-C samples.

Uniprot-KB code	Description (PTM)	Sum PEP Score	Coverage (%)	# Peptides	# Unique Peptides	# AAs	MW (kDa)	Score Sequest HT	Gene Symbol
P12429	Annexin A3	8.0	17	3	3	323	36.4	9.97	ANXA3
P02812	Basic salivary proline-rich protein 2	30.8	78	6	5	416	40.8	153.91	PRB2
Q9BYD5	Cornifelin	5.3	20	1	1	112	12.4	3.66	CNFN
P28325	Cystatin-D	16.5	39	3	3	142	16.1	28.86	CST5
P01036	Cystatin-S	10.5	32	3	2	141	16.2	16.71	CST4
P01037	Cystatin-SN	15.7	33	5	4	141	16.4	41.39	CST1
Q9UGM3	Deleted in malignant brain tumors 1 protein	25.4	26	7	7	2413	260.6	63.47	DMBT1
P81605	Dermcidin	16.8	32	5	5	110	11.3	53.08	DCD
P52907	F-actin-capping protein subunit alpha-1	2.9	6	1	1	286	32.9	6.88	CAPZA1
P04075	Fructose-bisphosphate aldolase A **(M_1-loss)**	34.6	36	8	8	364	39.4	70.87	ALDOA
P04406	Glyceraldehyde-3-phosphate dehydrogenase	24.6	28	5	5	335	36	58.12	GAPDH
P15515	Histatin-1	4.4	37	2	2	57	7	6.41	HTN1
P00338	L-lactate dehydrogenase A chain **(M_1-loss +N-term- Acetyl**)	8.8	14	3	2	332	36.7	18.27	LDHA
P02788	Lactotransferrin	3.0	3	1	1	710	78.1	6.93	LTF
P30740	Leukocyte elastase inhibitor	17.1	16	5	5	379	42.7	17.55	SERPINB1
Q9HC84	Mucin-5B	10.3	3	3	3	5762	596	26.28	MUC5B
Q8TAX7	Mucin-7	6.4	9	2	2	377	39.1	9.87	MUC7
P00558	Phosphoglycerate kinase 1	21.4	27	7	7	417	44.6	23.88	PGK1
P01833	Polymeric immunoglobulin receptor	41.3	22	13	13	764	83.2	101.89	PIGR
P06702	Protein S100-A9	16.6	54	4	4	114	13.2	35.13	S100A9
P29508	Serpin B3	5.8	4	1	1	390	44.5	4.93	SERPINB3
P02814	Submaxillary gland androgen-regulated protein 3B	8.3	66	2	2	79	8.2	132.53	SMR3B
Q6UWP8	Suprabasin	7.4	15	2	2	590	60.5	9.25	SBSN
P29401	Transketolase	3.7	3	1	1	623	67.8	4.6	TKT
P07951	Tropomyosin beta chain	14.4	26	5	2	284	32.8	17.66	TPM2
P08670	Vimentin	3.9	6	2	2	466	53.6	6.93	VIM

Table S5. Main MS data concerning the identification of proteins/peptides of the multi-protein complex (mPC) immunoprecipitated with cystatin D-C_{26} from salivary pools of SM+C samples. MS data corresponding to the two replicates (SM+C1 and SM+C2) are reported.

					SM+C1 Co-IP					SM+C2 Co-IP				
**Uniprot-KB code**	**Description (PTM)**	**Gene**	**# AAs**	**MW (kDa)**	**Sum PEP Score**	**Cov. (%)**	**# Pept.**	**# Unique Pept.**	**Score Sequest HT**	**Sum PEP Score**	**Cov. (%)**	**# Pept.**	**# Unique Pept.**	**Score Sequest HT**
P04083	Annexin A1	ANXA1	346	39	117.9	66	26	26	235.4	107.6	53	22	22	196.5
P02812	Basic salivary proline-rich protein 2	PRB2	416	41	11.2	59	4	3	24.7	6.2	46	3	3	9.7
P31944	Caspase-14	CASP14	242	28	4.6	5	2	2	4.2	2.8	5	1	1	1.7
Q9BYD5	Cornifelin	CNFN	112	12	4.7	20	1	1	4.1	5.6	20	1	1	3.6
P28325	Cystatin-D	CST5	142	16	6.8	29	2	2	17.9	6.4	29	2	2	10.3
P01037	Cystatin-SN	CST1	141	16	7.4	33	3	2	10.9	10.9	33	3	2	15.5
Q9UGM3	Deleted in malignant brain tumors 1 protein	DMBT1	2413	261	18.5	21	5	5	32.0	27.3	23	6	6	48.8
P15924	Desmoplakin	DSP	2871	332	32.3	7	13	13	36.2	27.8	6	12	12	57.2
P20930	Filaggrin	FLG	4061	435	3.4	5	2	2	7.6	5.9	5	3	3	7.5
P04075	Fructose-bisphosphate aldolase A **(M_1-loss)**	ALDOA	364	39	37.0	32	11	11	36.2	19.1	23	8	8	27.0
Q86YZ3	Hornerin	HRNR	2850	282	157.1	31	34	34	323.2	132.1	27	26	26	252.5
P30740	Leukocyte elastase inhibitor	SERPINB1	379	43	16.1	15	4	4	16.4	9.9	10	3	3	16.3
Q9HC84	Mucin-5B	MUC5B	5762	596	10.1	2	3	3	9.8	2.5	1	1	1	3.8
P00558	Phosphoglycerate kinase 1	PGK1	417	45	1.7	4	1	1	0.0	1.7	4	1	1	2.3
P01833	Polymeric immunoglobulin receptor	PIGR	764	83	30.9	17	8	8	39.1	34.8	16	9	9	69.5
P12273	Prolactin-inducible protein	PIP	146	17	7.8	14	3	3	22.5	9.0	14	3	3	26.6
P07237	Protein disulfide-isomerase	P4HB	508	57	2.0	4	1	1	2.9	6.1	5	2	2	6.0
P06702	Protein S100-A9	S100A9	114	13	11.2	53	4	4	23.3	10.8	29	4	4	16.3
P29508	Serpin B3	SERPINB3	390	45	8.7	6	2	2	13.9	6.3	9	3	3	8.0
P02814	Submaxillary gland androgen-regulated protein 3B	SMR3B	79	8	5.3	66	2	2	17.2	3.0	27	1	1	34.6
P68371	Tubulin beta-4B chain	TUBB4B	445	50	9.5	12	4	4	7.3	9.5	12	4	4	7.3
P25311	Zinc-alpha-2-glycoprotein	AZGP1	298	34	1.9	3	1	1	6.6	2.1	3	1	1	8.9

Table S6. Main MS data concerning the identification of proteins/peptides of the multi-protein complex (mPC) immunoprecipitated with cystatin D-C_{26} from salivary pools of SM samples. MS data corresponding to the two replicates (SM1 and SM2) are reported.

**Uniprot-KB code**	**Description (PTM)**	**Gene**	**# AAs**	**MW (kDa)**	**Sum PEP Score**	**Cov. (%)**	**# Pept.**	**# Unique Pept.**	**Score Sequest HT**	**Sum PEP Score**	**Cov. (%)**	**# Pept.**	**# Unique Pept.**	**Score Sequest HT**
					**SM1 Co-IP**					**SM2 Co-IP**				
P31947	14-3-3 protein sigma	SFN	248	28	99.3	85	17	15	104.9	6.9	25	3	3	11.3
P05141	ADP/ATP translocase 2 **(M_1-loss +N-term.-Acetyl.)**	SLC25A5	298	32.8	22.882	16	5	2	24.43	4.9	8.0	1.0	1.0	10.6
P04083	Annexin A1 **(M_1-loss +N-term.-Acetyl. Phospho-S_{37})**	ANXA1	346	39	298.6	88	44	44	563.4	139.4	69	33	33	226.8
P12429	Annexin A3	ANXA3	323	36	12.0	18	3	3	6.1	10.6	18	3	3	9.9
P20160	Azurocidin	AZU1	251	27	21.2	24	4	4	17.6	9.5	14	3	3	11.6
P04080	Cystatin-B **(N-term.-Acetyl.)**	CSTB	98	11	10.9	49	3	3	39.3	2.1	12	1	1	2.6
P28325	Cystatin-D	CST5	142	16	12.2	29	2	2	25.2	5.2	29	2	2	14.9
Q9UGM3	Deleted in malignant brain tumors 1 protein	DMBT1	2413	261	57.3	30	11	11	112.4	66.9	30	10	10	141.0
P15924	Desmoplakin	DSP	2871	332	70.8	13	27	27	85.4	17.8	3	7	7	34.9
P52907	F-actin-capping protein subunit alpha-1 **(M_1-loss +N-term.-Acetyl.)**	CAPZA1	286	33	9.9	15	3	3	5.8	3.8	5	1	1	2.5
Q01469	Fatty acid-binding protein 5 **(M_1-loss +N-term.-Acetyl.)**	FABP5	135	15	2.7	7	1	1	34.9	2.4	7	1	1	30.8
P04075	Fructose-bisphosphate aldolase A **(M_1-loss)**	ALDOA	364	39	44.0	45	11	11	42.0	24.0	24	7	7	20.6
P15104	Glutamine synthetase	GLUL	373	42	11.8	6	2	2	4.0	3.7	5	1	1	5.8
P14923	Junction plakoglobin	JUP	745	82	65.8	35	18	18	116.5	11.9	12	6	6	14.7
P00338	L-lactate dehydrogenase A chain **(M_1-loss +N-term.-Acetyl.)**	LDHA	332	37	15.2	18	5	5	36.4	9.1	17	4	4	16.3
P02788	Lactotransferrin	LTF	710	78	68.2	42	21	21	82.8	12.6	10	5	5	11.1
P30740	Leukocyte elastase inhibitor	SERPINB1	379	43	42.1	27	8	8	40.2	12.3	16	4	4	10.2
Q9HC84	Mucin-5B	MUC5B	5762	596	10.5	2	3	3	9.6	10.4	2	4	4	12.1
P59665	Neutrophil defensin 1	DEFA1; DEFA1B	94	10	6.8	20	3	3	42.9	4.9	20	3	3	16.9
P00558	Phosphoglycerate kinase 1	PGK1	417	45	25.8	23	6	6	14.4	14.7	21	5	5	12.2
Q13835	Plakophilin-1	PKP1	747	83	28.2	14	8	8	33.9	2.6	1	1	1	4.4

Uniprot-KB code	Description (PTM)	Gene	# AAs	MW (kDa)	Sum PEP Score	Cov. (%)	# Pept.	# Unique Pept.	Score Sequest HT	Sum PEP Score	Cov. (%)	# Pept.	# Unique Pept.	Score Sequest HT
					SM1 Co-IP					SM2 Co-IP				
P01833	Polymeric immunoglobulin receptor	PIGR	764	83	126.3	42	26	26	179.6	36.1	14	8	8	54.7
P12273	Prolactin-inducible protein	PIP	146	17	19.7	34	5	5	28.1	20.8	53	7	7	36.3
P05109	Protein S100-A8	S100A8	93	11	48.2	83	10	10	124.8	12.9	59	4	4	26.7
P06702	Protein S100-A9	S100A9	114	13	31.5	61	8	8	38.8	19.1	59	5	5	19.2
P29508	Serpin B3	SERPINB3	390	44.5	17.1	20	6	4	11.7	2.4	4	1	1	2.5
P29401	Transketolase	TKT	623	68	3.0	3	1	1	2.4	10.2	7	3	3	10.7
P62987	Ubiquitin-60S ribosomal protein L40	UBA52	128	15	6.4	16	2	2	10.8	2.1	13	1	1	6.9
P21796	Voltage-dependent anion-selective channel protein 1	VDAC1	283	31	9.8	20	3	3	7.5	2.7	6	1	1	2.1

Table S7. Biological processes and cellular localization concerning the proteins/ peptides identified as cystatin D-C_{26} interactors, provided by PD software based on the GO term databases.

Uniprot-KB code	Name	Biological Process	Cellular Component
		Interactors characterized in Ctrls and patients	
P31947	**14-3-3 protein sigma**	Cell growth; coagulation; defense response; development; metabolic process; formation of multiprotein complexes	Cytoplasm; nucleus, secreted
P05141	**ADP/ATP translocase 2**	Cell growth; cellular homeostasis; conjugation; defense response; metabolic process	Mitochondrion inner membrane
Q9BYD5	**Cornifelin**	Defense response; cell envelope formation of epithelia	Cytoplasm; cell envelope
P28325	**Cystatin D**	Inhibitor of cathepsins; innate immunoresponse; cell antimigratory and antiproliferative activity	Secreted, cytoplasm, nucleus
P04080	**Cystatin B**	Inhibitor of cathepsins; innate immunoresponse; metabolic process	Secreted, cytoplasm, nucleus
P01036	**Cystatin S**	Oral cavity homeostasis; protease inhibition; metabolic process	Secreted
P01037	**Cystatin SN**	Protease inhibition; defense response; metabolic process	Secreted
Q9UGM3	**DMBT1**	Defense response; LPS binding; coagulation; conjugation; metabolic process	Secreted
P15924	**Desmoplakin**	Cell communication; cell growth; cell organization and biogenesis; cellular homeostasis; coagulation; defense response; metabolic process	Cell junction, cell membrane, membrane
P04075	**Fructose-bisphosphate aldolase A**	cell growth; cellular homeostasis; glucose metabolic processes	Cytoplasm
P02788	**Lactotransferrin**	Cell organization and biogenesis; coagulation; conjugation; defense response; metabolic process	Cytoplasm, nucleus, secreted
Q9HC84	**Mucin-5B**	Oral cavity homeostasis; defense response	Secreted
P01833	**PIgR**	Immune response; onjugation; development; metabolic process	Cell membrane, secreted
P12273	**PIP**	Cell organization and biogenesis; metabolic process	Secreted
P06702	**S100A9**	Inflammatory response; Cell communication; cell death; cell organization and biogenesis; cellular homeostasis; coagulation; defense response; metabolic process	Cell membrane, cytoplasm, cytoskeleton, secreted
P29508	**Serpin B3**	Cell death; metabolic process	Cytoplasm
		**Interactors characterized only in Ctrls**	
P63104	**14-3-3 protein zeta/delta**	Cell growth; cell organization and biogenesis; cellular homeostasis; conjugation; defense response; development; metabolic process; formation of multiprotein complexes	Cytoplasm
P59998	**ARPC4**	Actin-polymerization and cytoskeleton organization; cell growth	Cytoplasm, cytoskeleton, nucleus
P08311	**Cathepsin G**	Defense response; coagulation; inflammation; protease activity	Cytoplasm; lysosome; secreted; nucleus
O00299	**Chloride intracellular channel protein 1**	Cellular homeostasis; cell cycle regulation; conjugation; development; metabolic process	Transmembrane
P23528	**Cofilin-1**	Cell differentiation; cell growth; development; metabolic process; cytoskeleton organization	Cell membrane, cytoplasm, cytoskeleton, nucleus

Uniprot-KB code	Name	Biological Process	Cellular Component
O43240	**Kallikrein-10**	Cell differentiation; endopeptidase activity; cell organization and biogenesis	Secreted
P61626	**Lysozyme C**	Cellular homeostasis; defense response; coagulation; metabolic process	Secreted
P35232	**Prohibitin**	Cell growth; cell organization and biogenesis; cell proliferation; cellular homeostasis; coagulation; development; metabolic process; mitochondrial homeostasis and signal transduction; mitochondrial-mediated antiviral innate immunity	cytoplasm; cytoskeleton; endosome; membrane; mitochondrion; nucleus
P13489	**Ribonuclease inhibitor**	Cellular component movement; cellular homeostasis; metabolic process	Cytoplasm
		**Interactors characterized only in patients (SM, SM+C, SM-C)**	
P20160	**Azurocidin**	Cell organization and biogenesis; coagulation; defense response; development; metabolic process; LPS-binding	Membrane
P02812	**bPRP-2**	Oral cavity homeostasis; constitution of the protein pellicle; dietary tannin binding	Secreted
Q01469	**FABP5**	Cellular homeostasis; conjugation; metabolic process	Cytoplasm, nucleus, secreted, synapse
P15104	**Glutamine synthetase**	Cell differentiation; cell organization and biogenesis; cellular homeostasis; coagulation; defense response; metabolic process	Cell membrane, cytoplasm, endoplasmic reticulum, microsome, mitochondrion
P14923	**Junction plakoglobin**	Cell communication; cytoskeleton organization cell growth; defense response; metabolic process	Cell junction, cytoplasm, cytoskeleton, membrane
P30740	**Leukocyte elastase inhibitor**	Protease inhibition; immune response; cell differentiation; metabolic process	Cytoplasm, endosome, lysosome, secreted
P59665	**Neutrophil defensin 1**	Defense response; coagulation; development; metabolic process	Secreted
P02814	**P-B peptide**	Cellular response to LPS; Metabolic process	Secreted
Q13835	**Plakophilin-1**	Cell communication; cytoskeleton organization; cell growth; development; metabolic process	Cell junction, nucleus
P00558	**Phosphoglycerate kinase 1**	cell organization and biogenesis; cellular homeostasis; coagulation; glucose metabolic processes	Cytoplasm
	**S100A8**	Inflammatory response; Cell communication; cell death; cell organization and biogenesis; cellular homeostasis; coagulation; defense response; metabolic process	Cytoplasm; secreted
P62987	**Ubiquitin-60S ribosomal protein L40**	Cell organization and biogenesis; ribosomal protein synthesis; cellular homeostasis; metabolic process	Cytoplasm, nucleus
P21796	**Voltage-dependent anion-selective channel protein 1**	Cell death; cellular homeostasis; coagulation; conjugation; metabolic process	Mitochondrion outer membrane, cell membrane
		**Interactors characterized only in SM+C patients**	
P04083	**Annexin A1**	Cell death; cell growth; cell organization and biogenesis; cellular homeostasis; coagulation; conjugation; defense response; development; metabolic process; prostaglandin synthesis and regulation	Cell membrane, cytoplasm, cytoplasmic vesicle, endosome, nucleus, secreted
P31944	**Caspase-14**	Cell organization and biogenesis; apoptosis; defense response; metabolic process	Cytoplasm; nucleus

Uniprot-KB code	Name	Biological Process	Cellular Component
P20930	**Filaggrin**	cell organization and biogenesis; cellular homeostasis; defense response; formation of the cornified envelope	Cytoplasmic granule
Q86YZ3	**Hornerin**	Cell growth; defense response; cornified cell envelope	Cytoplasmic granule
P68371	**Tubulin beta-4B chain**	Cell differentiation; cell growth; coagulation	Cytoplasm, cytoskeleton, microtubule
P25311	**Zinc-alpha-2-glycoprotein**	Cell communication; cellular component movement; cellular homeostasis; conjugation; lipids metabolism	Secreted
		**Interactors characterized only in SM-C patients**	
P12429	**Annexin A3**	Coagulation; conjugation; defense response; metabolic process	Cytoplasm, extracellular exosome, phagocytic vesicle membrane
P81605	**Dermcidin**	Cell organization and biogenesis; defense response; coagulation; metabolic process	Secreted, transmembrane
P52907	**F-actin-capping protein subunit alpha-1**	Cell growth; cytoskeleton organization	Cytoplasm, cytoskeleton
P04406	**GAPDH**	cell growth; cell organization and biogenesis; cellular homeostasis; coagulation; glucose metabolic processes	Cytoplasm, cytoskeleton, membrane, nucleus
P15515	**Hst-1**	Coagulation; defense response; metabolic process	Secreted
P00338	**LDH**	Cellular homeostasis; coagulation; defense response; metabolic process; Cori cycle	Cytoplasm
Q8TAX7	**Mucin-7**	Defense response; metabolic process	Secreted
P07237	**PDI**	Cell organization and biogenesis; cellular homeostasis; coagulation; development; metabolic process	Cell membrane, endoplasmic reticulum
Q6UWP8	**Suprabasin**		Cytoplasm, exosome
P29401	**Transketolase**	cellular homeostasis; metabolic process; pentose phosphate metabolism	Cytoplasm, extracellular exosome, nucleus, peroxisome
P07951	**Tropomyosin beta chain**	Cell growth; metabolic process	Cytoplasm, cytoskeleton
P08670	**Vimentin**	Cell growth; coagulation; defense response; development; metabolic process	Cell membrane, cytoplasm, cytoskeleton, nucleus
